# The Opportunistic Pathogen *Propionibacterium acnes*: Insights into Typing, Human Disease, Clonal Diversification and CAMP Factor Evolution

**DOI:** 10.1371/journal.pone.0070897

**Published:** 2013-09-13

**Authors:** Andrew McDowell, István Nagy, Márta Magyari, Emma Barnard, Sheila Patrick

**Affiliations:** 1 Centre for Infection and Immunity, School of Medicine, Dentistry and Biomedical Sciences, Queen's University, Belfast, United Kingdom; 2 Institute of Biochemistry, Biological Research Centre of the Hungarian Academy of Sciences, Szeged, Hungary; Beijing Institute of Microbiology and Epidemiology, China

## Abstract

We previously described a Multilocus Sequence Typing (MLST) scheme based on eight genes that facilitates population genetic and evolutionary analysis of *P. acnes*. While MLST is a portable method for unambiguous typing of bacteria, it is expensive and labour intensive. Against this background, we now describe a refined version of this scheme based on two housekeeping (*aroE*; *guaA*) and two putative virulence (*tly*; *camp2*) genes (MLST_4_) that correctly predicted the phylogroup (IA_1_, IA_2_, IB, IC, II, III), clonal complex (CC) and sequence type (ST) (novel or described) status for 91% isolates (n = 372) via cross-referencing of the four gene allelic profiles to the full eight gene versions available in the MLST database (http://pubmlst.org/pacnes/). Even in the small number of cases where specific STs were not completely resolved, the MLST_4_ method still correctly determined phylogroup and CC membership. Examination of nucleotide changes within all the MLST loci provides evidence that point mutations generate new alleles approximately 1.5 times as frequently as recombination; although the latter still plays an important role in the bacterium's evolution. The secreted/cell-associated ‘virulence’ factors *tly* and *camp2* show no clear evidence of episodic or pervasive positive selection and have diversified at a rate similar to housekeeping loci. The co-evolution of these genes with the core genome might also indicate a role in commensal/normal existence constraining their diversity and preventing their loss from the *P. acnes* population. The possibility that members of the expanded CAMP factor protein family, including *camp2*, may have been lost from other propionibacteria, but not *P. acnes*, would further argue for a possible role in niche/host adaption leading to their retention within the genome. These evolutionary insights may prove important for discussions surrounding *camp2* as an immunotherapy target for acne, and the effect such treatments may have on commensal lineages.

## Introduction

*Propionibacterium acnes* is an anaerobic, Gram-positive opportunistic pathogen that forms part of the normal microbiota on human skin and mucosal surfaces. The traditional view of this bacterium as a relatively harmless member of the normal human microbiota is now constantly challenged in the literature, with increasing descriptions of its role in human infections and clinical conditions. Although most notably linked to the inflammatory skin condition acne vulgaris [1], growing evidence now supports an important role in other human diseases, including infections of indwelling medical devices [2]–[4], and potential roles in the aetiology of sarcoidosis [5,6] and prostate cancer [7], [8]. Despite this, the fact that *P. acnes* is present in both the normal and disease states often frustrates attempts to assign a definite role in various human diseases via fulfilment of Koch's postulates.

In collaboration with the University of Warwick, UK, we previously described a Multilocus Sequence Typing (MLST) scheme and database for *P. acnes* that utilised seven housekeeping genes (MLST_7_; 3135 bp) [9]. The Warwick scheme, which was established to build upon our earlier epidemiological and phylogenetic studies of *P. acnes*, resolves isolates into major evolutionary clades, designated types IA_1_, IA_2_, IB, IC, II and III, which differ in their association with disease, production of putative virulence determinants, immunogenic and inflammatory properties, as well as biochemical, aggregative and morphological characteristics [7], [9]–[18]. We used this scheme to stratify isolates into sequence types (STs) within these statistically significant genetic groupings, thus facilitating studies of their association with different infections and cell surface antigens [9]. To provide a deeper level of resolution, primarily for the type IA_1_ phylogroup, we recently published an updated and expanded eight gene version of the scheme based on six housekeeping and two ‘putative virulence’ genes; the latter encode a proposed secreted haemolysin/cytotoxin (*tly*) and cell-associated Christie Atkins Munch Petersen (CAMP) homologue (4253 bp) [17]. The introduction of virulence genes into MLST schemes is now increasingly common as these loci are often more diverse and therefore provide greater discrimination as a result of positive or diversifying selection. Application of the scheme to a large collection of isolates from disparate clinical sources, as well as healthy skin, identified clonal complexes (CC) and associated lineages with an apparent heightened capacity to cause infection, and others that may be associated with a commensal existence and skin health. This was in keeping with the observations of Lomholt and Kilian [16].

Compared with gel-based typing schemes, such as pulsed-field gel electrophoresis or random amplification of polymorphic DNA (RAPD) methods, MLST is a highly portable technique that provides unambiguous genotype assignments and also provides data that is amenable for evolutionary and population genetic analysis. Furthermore, due to the greater number of informative nucleotide sites analysed, as well as the buffering effects that multiple loci provide against the distorting effects of recombination, MLST provides much deeper and more accurate population genetic data on *P. acnes* compared to previously described single locus phylotyping methods which have solely targeted the *tly* or *recA* gene [11], [15], [18]–[22]; this latter locus has been widely applied in bacterial systematics and has proven valuable for phylogenetic resolution of closely related taxa that show high 16S rDNA sequence identity [23]. While the MLST method has become the gold standard for bacterial typing, it is expensive, as well as labour intensive and time consuming. This has limited its widespread uptake by researchers and routine diagnostic laboratories. A scheme that could provide phylogenetically accurate genotyping information, but based on a significantly reduced number of loci would, therefore, be beneficial and attractive. In the first part of this paper we describe a refined four gene MLST scheme based on *aroE-guaA-tly-camp2* (MLST_4_) that provides valuable typing data for researchers, but with a significant reduction in costs, labour and time. The method should be particularly suitable for the analysis of large collections of isolates, enabling stratification of strains for further downstream study. We then develop this MLST theme by using our current multilocus sequence data to further understand the role of specific lineages in human health and disease. In the second part we provide insights into the roles played by mutation and recombination during clonal diversification and propose a probable model for CAMP factor homologue evolution in propionibacteria. These evolutionary-based studies are especially relevant to ongoing discussions concerning the use of CAMP factor 2 as a potential therapeutic target.

## Results and Discussion

### Levels of genetic diversity between MLST loci

The number of loci selected for MLST analysis depends on the particular epidemiological questions being asked and the minimum resolution necessary to provide the answer; this also has to be balanced against the time and expense required when characterising multiple isolates. Against this background, we examined what impact reducing the eight loci we routinely utilise for MLST would have on the discriminatory power of our scheme and whether it would still be possible to accurately determine the phylogroup, clonal complex (CC) and ST of isolates by cross-referencing a reduced allelic profile to the full profiles available in the growing *P. acnes* MLST database (http://pubmlst.org/pacnes/). If possible, this would have significant financial and practical implications for researchers wishing to analyse large numbers of *P. acnes* isolates.

At the onset of this specific study we had examined a total of 285 isolates by MLST_8_ resulting in the generation of 91 STs. Against this large collection of sequence data we determined the most diverse set of loci within our scheme based on a range of criteria detailed in Table 1. The number of alleles per locus ranged from 10 (*lepA*) to 33 (*camp2*), while the number of polymorphic sites ranged from 16 (3.54%; *lepA*) to 79 (9.82%; *camp2*) and nucleotide diversity by locus varied from 1.00% (*atpD*) to 2.60% (*camp2*). The discriminatory index (*D*) was the lowest for *atpD* (42.3%) and the highest for *camp2* (83.7%). While the *tly* and *camp2* genes had the highest number of alleles due to greater sequence lengths, the % polymorphic sites and π values were not significantly different from the housekeeping loci providing evidence that all the genes were diversifying at approximately similar rates (Table 1).

**Table 1:** Loci characteristics and discriminatory power of MLST schemes based on four (MLST_4_) and eight (MLST_8_) genes.

Loci	Size (bp)	Alleles	No. Polymorphic sites	% polymorphic sites	p-distance^a^	π^b^	θ^c^	*d_N_/d_S_*	Tajima's*D* test^d^	% discriminatory index (95% confidence intervals)
*aroE*	424	19	38	8.96	2.22%	0.021	0.026	0.147	−0.712	47.1 (40.1–54.1)
*atpD*	453	13	22	4.86	1.00%	0.010	0.016	0.150	−1.487	42.3 (35.6–49.0)
*gmk*	400	12	20	5.00	1.40%	0.014	0.016	0.105	−0.725	43.8 (37.1–50.5)
*guaA*	493	18	32	6.49	2.30%	0.022	0.019	0.025	0.545	70.6 (66.5–74.7)
*lepA*	452	10	16	3.54	1.20%	0.012	0.013	0.107	−0.351	45.5 (38.6–52.5)
*sodA*	450	14	21	4.67	1.40%	0.014	0.015	0.063	−0.244	69.0 (64.4–73.6)
*tly*	777	23	49	6.31	1.70%	0.016	0.018	0.299	−0.337	79.5 (76.8–82.3)
*camp2*	804	33	79	9.82	2.60%	0.026	0.025	0.295	0.066	83.7 (81.3–86.0)
MLST_4_	2498	–	–	–	1.89%	–	–	–	–	93.0 (91.3–94.5)
MLST_8_	4253	–	–	–	1.50%	–	–	–	–	94.0 (92.3–95.3)

a p-distance determined using Kimura Two parameter nucleotide substitution model.

b Nucleotide diversity per site.

c Average number of nucleotide differences per site.

d p>0.1 for all Tajima's D test results.

### Discriminatory power of the MLST_4_ scheme for genotyping of *P. acnes*

**Figure 1:**
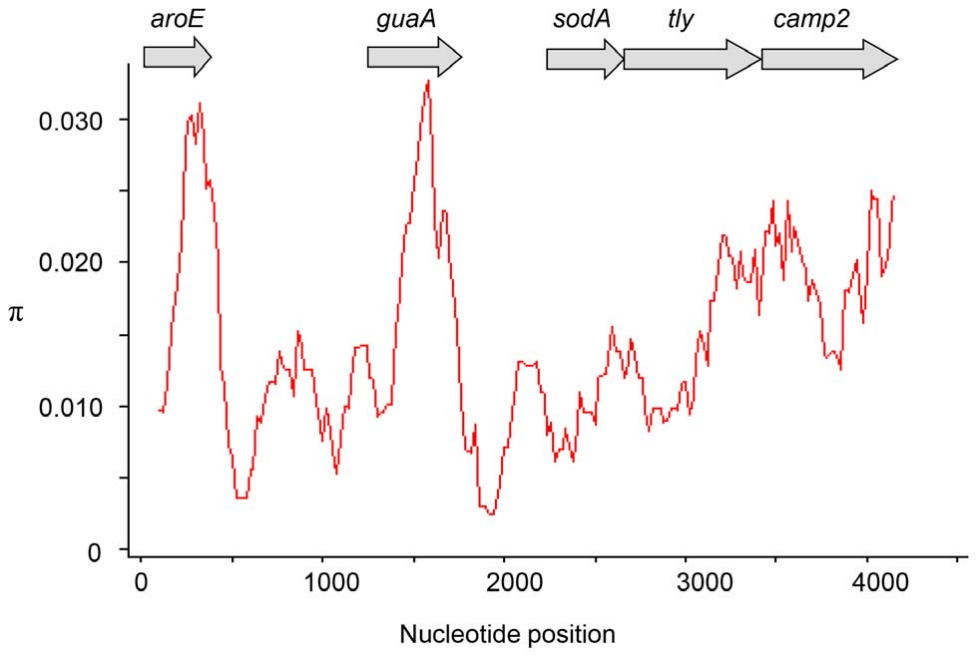
Sliding window analysis of nucleotide diversity (π) within the concatenated sequence of all eight MLST loci (4253 bp). Analysis was carried out using a window size of 200(*aroE, guaA, sodA, tly* and *camp2*) selected for further analysis are indicated.

On the basis of these initial analyses, five loci were selected for further investigation; these were *aroE*, *guaA*, *sodA*, *tly* and *camp2* (Figure 1). With the sequence information available from the previous analysis of 285 isolates [17], we initially investigated a four locus MLST approach based on a combination of *aroE-guaA-tly-camp2* (MLST_4_; 2498 bp) for the discrimination of *P. acnes* isolates. To prevent confusion, the same ST numbering of unique genotypes was maintained between schemes. A total of 75 distinct STs were resolved with an overall *D* value of 93% (95% CI 91.3–94.5) (Table 1; Table S1). With the eight gene scheme (MLST_8_) a total of 91 STs were resolved with a *D* value of 94% (95% CI 92.3–95.3) (p<0.001) (Table 1; Table S1). The overall concordance between the two methods as measured by the adjusted Rand value was 91.8% (95% CI 87–96.7). We also compared these results with a four locus scheme based on a combination of *guaA-sodA-tly-camp2* (2524 bp). With these loci, only 65 distinct STs were differentiated with an overall *D* value of 91% (95% CI 89.3–93.1). This reduced discriminatory index value was significantly different from both the MLST_4_ (p<0.001) and MLST_8_ schemes (p<0.001). Rand values between this combination of alleles and MLST_8_ were 81.2% (95% CI 73.8–88.7) and between both MLST_4_ methods 89.1% (95% CI 83.1–95.1). On this basis, further analyses were only carried out with the *aroE-guaA-tly-camp2* scheme.

We further examined the MLST_4_ method against the *P. acnes* MLST scheme adopted by Lomholt and Kilian [16] at Aarhus University, Denmark and based on nine housekeeping genes (MLST_9_; all different from our scheme). To achieve this, we examined a subset of 86 *P. acnes* isolates from our MLST collection for which Aarhus MLST data was available. These included 72 isolates from the Human Microbiome Project (HMP) and other sequencing programmes, along with 14 Danish isolates described in the original Aarhus MLST publication [16]. Results from the MLST_4_ and MLST_9_ methods were similar with both schemes discriminating a total of 27 STs, with *D* values of 91% (95% CI 87.5–93.6) and 93% (CI 91.2–95.6%), respectively (p = 0.105). With the MLST_8_ method, a total of 32 STs were resolved with a *D* value of 93% (CI 91.3–95.9%) (p = 0.058 vs MLST_4_ and p = 0.353 vs MLST_9_). While MLST_8_ appears to demonstrate higher levels of resolution overall when compared to the MLST_9_ method, the two schemes are essentially concordant in respect to the clustering of isolates into CCs, although some differences in the resolution of particular lineages within CCs exist [17].

By cross-referencing the allelic profile for each of the 75/91STs (82%) resolved by MLST_4_ to the full allelic profiles currently available within the *P. acnes* MLST database (http://pubmlst.org/pacnes/), it was possible to accurately determine the phylogroup (IA_1_, IA_2_, IB, IC, II and III) and CC/singleton identity of the isolates (Table S1). Even for the 16 STs that could not be fully resolved using the MLST_4_ approach, their allelic profile still accurately predicted phylogroup and CC membership (Table S1). For these STs, the MLST_4_ method still generated important phylogenetic data.

### Comparison of MLST analysis with 16S rDNA ribotyping methods

We also compared the discriminatory power of our MLST methods with the results obtained upon 16S rDNA-based ribotyping analysis, which has recently been described as a useful method to distinguish major *P. acnes* lineages [24]; to do this, we compared 81 isolates from the HMP. Analysis of this isolate cohort by MLST_8_ resolved a total of 35 STs with a *D* value of 93% (CI 89.5–95.7%), and by MLST_4_ a total of 32 STs with a *D* value of 92% (CI 88.6–95.1%) (p = 0.139). In comparison, sequence analysis of the entire 16S rDNA gene revealed only 10 ribotypes with a *D* value of 81% (CI 75.2%–86.1%). As expected, 16S rDNA-based ribotyping had significantly reduced resolving power when compared to MLST analysis based on multiple protein-encoding genes (p<0.001), which generally diverge at a faster rate. This is illustrated by the overlap of ribotype 1 between strains from the type IA_1_ and IB divisions; and ribotype 5 between strains from the IA_1_ and IC phylogroups. Furthermore, inter-division (I, II and III) ribotypes, unlike equivalent *recA* alleles, only differ by a very small number of polymorphic sites. The clear differences between both typing approaches must be balanced, however, against the practicalities of analysing only one gene (16S rDNA) versus multiple genes (MLST). The method adopted will therefore depend on the degree of resolution required for the epidemiological issues being addressed.

### New *aroE* amplification and sequencing primers for MLST

Over the course of our previous MLST studies we have observed that the PCR primers described for *aroE*, and originally designed against the then only available whole genome sequence of *P. acnes* represented by strain KPA171202 (type IB, ST5, CC5) [25], can give poor amplification of this gene from type II strains. Furthermore, we have also found that *aroE* nucleotide sequences produced using the reverse sequencing primer often produce reduced quality reads compared to those obtained with the forward sequencing primer. To investigate these issues further, we aligned complete *aroE* gene sequences now available for *P. acnes* isolates (via HMP) representing the major phylogroups (IA_1_, IA_2_, IB, IC, II and III) and found mismatches very close to the terminal 3′ ends for both the forward and reverse amplification primers when compared to type II strains (Figure S1). The reverse sequencing primer also had a T/G mismatch at the penultimate base of the 3′ end when aligned against type II strains (not shown). We therefore designed new forward and reverse primers that flank the 5′ and 3′ ends of the gene and used them for both amplification and sequencing (see Materials and Methods). The primers produce excellent amplicon yields and sequencing reads from all the phylogroups (data not shown).

### MLST_4_ genotyping in practice

**Figure 2:**
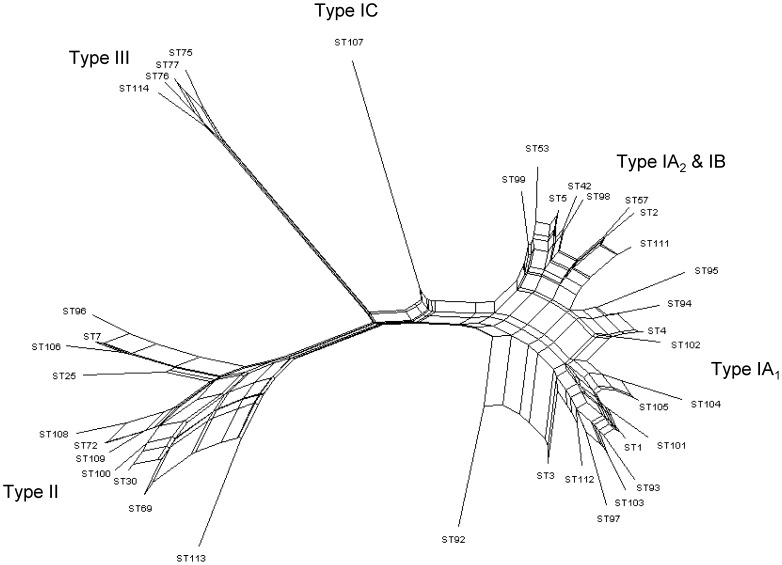
Neighbour-net splits graph of allelic profiles from all 38 STs identified upon MLST analysis of 87 *P. acnes* isolates. STs representing all phylogroups were identified from this cohort of isolates. Parallelogram structures indicative of recombination events are evident within the major type I and II divisions.

To investigate how the MLST_4_ approach would work in practice, we analysed a new batch of *P. acnes* isolates (n = 87) recovered from a wide range of different clinical sources (Table 2) and compared the results to those obtained after MLST_8_ analysis (Table 3). The assignment of ST numbers based on four gene allelic profiles was as outlined in Table S1. A total of 28 STs were resolved using the MLST_4_ method and 38 STs by MLST_8_ (Figure 2; Table 3). Overall, we identified 35 (40%) type IA_1_ isolates_,_ four (5%) type IA_2_, 27 (31%) type IB, one (1%) type IC, 15 (17%) type II and five (6%) type III (Figure 2; Table 2). With the MLST_4_ scheme, a total of 15 isolates were correctly highlighted as a new ST based on the identification of a novel allele for one of the four loci analysed (Table 3). No other novel alleles were identified when analysis was extended to the *atpD*, *gmk*, *lepA* and *sodA* gene sequences for these isolates. With MLST_8_, a total of 22 novel STs were discovered; seven of these new STs were not identified by MLST_4_ as the novel alleles resided on the *atpD*, *gmk*, *lepA* and *sodA* loci (Table 3). In total, the genotype status of 74/87 (85%) isolates was correctly identified as either a novel ST or an already described ST (along with phylogroup and CC) by cross-referencing of the MLST_4_ allelic profiles to full profiles available in the database (Table 3). In 12/13 cases where the specific ST was not correctly identified, or novel genotype highlighted, the MLST_4_ allelic profiles still correctly determined phylogroup and CC membership (Table 3). Only one isolate, 5–1–3, was not accurately assigned to either CC or ST, but was correctly placed within phylogroup IC (Table 3).

**Table 2:** Summary of phylogroups and their source for 87 isolates analysed by MLST in this study.

	Phylogroup						
Source	Type IA_1_	Type IA_2_	Type IB	Type IC	Type II	Type III	Total
Normal skin	2	1	8	–	3	5	19
Acne lesion	16	3	–	1	4	–	24
Blood	6	–	3	–	6	–	15
Wound	3	–	4	–	–	–	7
Abscess	3	–	1	–	–	–	4
Conjunctiva	–	–	2	–	1	–	3
Surgical	–	–	2	–	1	–	3
Intraabdominal	1	–	1	–	–	–	2
Pleural aspirate	1	–	1	–	–	–	2
Oral	1	–	1	–	–	–	2
Joint aspirate	–	–	1	–	–	–	1
Heart implant	1	–	–	–	–	–	1
Otitis media	–	–	1	–	–	–	1
Intrauterine	–	–	1	–	–	–	1
Appendix	–	–	1	–	–	–	1
Cystitis	1	–	–	–	–	–	1
Total	35 (40%)	4 (5%)	27 (31%)	1 (1%)	15 (17%)	5 (6%)	87 (100%)

**Table 3:** MLST results generated for 87 *P. acnes* isolates upon analysis with the four (MLST_4_) and eight (MLST_8_) gene schemes.

			MLST_4_		MLST_8_		
Isolate	Source	Region	Allelic profile	ST/CC	Allelic profile	ST/CC	Phylogroup
KF16	Acne	Hungary	1–3–1–1	ST1/CC1	1–1–1–3–1–1–1–1	ST1/CC1	IA_1_
HDN-1	Acne	UK	1–3–1–1	ST1/CC1	1–1–1–3–1–1–1–1	ST1/CC1	IA_1_
IT100EA	Acne	UK	1–3–1–1	ST1/CC1	1–1–1–3–1–1–1–1	ST1/CC1	IA_1_
SP64E	Acne	UK	1–3–1–1	ST1/CC1	1–1–1–3–1–1–1–1	ST1/CC1	IA_1_
55835	Cystitis	Hungary	1–3–1–1	ST1/CC1	1–1–1–3–1–1–1–1	ST1/CC1	IA_1_
3632	Blood	Hungary	1–3–1–1	ST1/CC1	1–1–1–3–1–1–1–1	ST1/CC1	IA_1_
4579	Skin	Hungary	1–3–1–1	ST1/CC1	1–1–1–3–1–1–1–1	ST1/CC1	IA_1_
12604	Pleural aspirate	Hungary	1–3–1–1	ST1/CC1	1–1–1–3–1–1–1–1	ST1/CC1	IA_1_
16634	Acne	Hungary	1–3–1–1	ST1/CC1	1–1–1–3–1–1–1–1	ST1/CC1	IA_1_
63006	Acne	Hungary	1–3–1–1	ST1/CC1	1–1–1–3–1–1–1–1	ST1/CC1	IA_1_
19107	Abscess	Hungary	1–3–1–1	ST1/CC1	1–1–1–3–1–1–1–1	ST1/CC1	IA_1_
20550	Intraabdominal sample	Hungary	1–3–1–1	ST1/CC1	1–1–1–3–1–1–1–1	ST1/CC1	IA_1_
M43	Blood	Hungary	1–3–1–1	ST1/CC1	1–1–1–3–1–1–1–1	ST1/CC1	IA_1_
50460	Acne	Hungary	1–3–1–1	ST1/CC1	1–1–1–3–1–1–1–1	ST1/CC1	IA_1_
64745	Abscess	Hungary	1–3–1–1	ST1/CC1	1–1–1–3–1–1–1–1	ST1/CC1	IA_1_
2–2–1	Acne	France	1–3–1–1	ST1/CC1	1–1–1–3–1–1–1–1	ST1/CC1	IA_1_
2–4–6	Skin	France	1–3–1–1	ST1/CC1	1–1–1–3–1–1–1–1	ST1/CC1	IA_1_
1–1–2	Acne	France	1–5-8–2	ST2/CC2	1–1–1-5–1–4–8–2	ST2/CC2	IA_2_
1–3–2	Acne	France	1–5–8–2	ST2/CC2	1–1–1–5–1–4–8–2	ST2/CC2	IA_2_
15614	Blood	Hungary	1–3–2–2	ST3/CC3	1–1–1–3–1–1–2–2	ST3/CC3	IA_1_
26048	Blood	Hungary	1–3–2-2	ST3/CC3	1–1–1–3–1–1–2–2	ST3/CC3	IA_1_
A10	Acne	Hungary	1–3–8–6	ST4/CC4	1–1–1–3–1–1–8–6	ST4/CC4	IA_1_
82964	Wound	Hungary	1–3–8–6	ST4/CC4	1–1–1–3–1–1–8–6	ST4/CC4	IA_1_
94167	Abscess	Hungary	1–3–8–6	ST4/CC4	1–1–1–3–1–1–8–6	ST4/CC4	IA_1_
28585	Blood	Hungary	1–3–8–6	ST4/CC4	1–1–1–3–1–1–8–6	ST4/CC4	IA_1_
6–2–4	Acne	France	1–3–8–6	ST4/CC4	1–1–1–3–1–1–8–6	ST4/CC4	IA_1_
89071	Wound	Hungary	1–4–8–6	ST5/CC5	1–1–1–4–1–4–8–6	ST5/CC5	IB
96088	Skin	Hungary	1–4–8–6	ST5/CC5	1–1–1–4–1–4–8–6	ST5/CC5	IB
102778	Joint aspirate	Hungary	1–4–8–6	ST5/CC5	1–1–1–4–1–4–8–6	ST5/CC5	IB
1945	Pleural aspirate	Hungary	1–4–8–6	ST5/CC5	1–1–1–4–1–4–8–6	ST5/CC5	IB
4837	Surgical sample	Hungary	1–4–8–6	ST5/CC5	1–1–1–4–1–4–8–6	ST5/CC5	IB
10738	Abscess	Hungary	1–4–8–6	ST5/CC5	1–1–1–4–1–4–8–6	ST5/CC5	IB
14179	Blood	Hungary	1–4–8–6	ST5/CC5	1–1–1–4–1–4–8–6	ST5/CC5	IB
16703	Otitis media	Hungary	1–4–8–6	ST5/CC5	1–1–1–4–1–4–8–6	ST5/CC5	IB
20527	Blood	Hungary	1–4–8–6	ST5/CC5	1–1–1–4–1–4–8–6	ST5/CC5	IB
23766	Wound	Hungary	1–4–8–6	ST5/CC5	1–1–1–4–1–4–8–6	ST5/CC5	IB
24728	Wound	Hungary	1–4–8–6	ST5/CC5	1–1–1–4–1–4–8–6	ST5/CC5	IB
45808	Intrauterine device	Hungary	1–4–8–6	ST5/CC5	1–1–1–4–1–4–8–6	ST5/CC5	IB
51056	Intraoral sample	Hungary	1–4–8–6	ST5/CC5	1–1–1–4–1–4–8–6	ST5/CC5	IB
58828	Conjunctiva secretion	Hungary	1–4–8–6	ST5/CC5	1–1–1–4–1–4–8–6	ST5/CC5	IB
57826	Conjunctiva secretion	Hungary	1–4–8–6	ST5/CC5	1–1–1–4–1–4–8–6	ST5/CC5	IB
61489	Appendicitis	Hungary	1–4–8–6	ST5/CC5	1–1–1–4–1–4–8–6	ST5/CC5	IB
1–3–1	Skin	France	1–4–8–6	ST5/CC5	1–1–1–4–1–4–8–6	ST5/CC5	IB
2–4–5	Skin	France	1–4–8–6	ST5/CC5	1–1–1–4–1–4–8–6	ST5/CC5	IB
4–2–8	Skin	France	1–4–8–6	ST5/CC5	1–1–1–4–1–4–8–6	ST5/CC5	IB
4–4–1	Skin	France	1–4–8–6	ST5/CC5	1–1–1–4-1–4–8–6	ST5/CC5	IB
6–3–1	Skin	France	1–4–8–6	ST5/CC5	1–1–1–4–1–4–8–6	ST5/CC5	IB
7–2–6	Skin	France	1–4–8–6	ST5/CC5	1–1–1–4–1–4–8–6	ST5/CC5	IB
72995	Surgical sample	Hungary	15–4–10–10	ST7/CC6	15–4–2–4–2–3–10–10	ST7/CC6	II
44073	Blood	Hungary	15–4–10–10	ST7/CC6	15–4–2–4–2-3–10–10	ST7/CC6	II
65763	Blood	Hungary	15–4–10–10	ST7/CC6	15–4–2–4–2–3–10–10	ST7/CC6	II
65311^a^	Blood	Hungary	17–4–10-10	ST6/CC6	17–9–2–4–2–3–10–10	ST25/CC6	II
76618	Blood	Hungary	17–4–10–12	ST30/CC72	17–4–2–4–2–6–10–12	ST30/CC72	II
6167	Blood	Hungary	17–4–10–12	ST30/CC72	17–4–2–4–2–6–10–12	ST30/CC72	II
74873	Blood	Hungary	1–4–8–21	ST42/CC5	1–1–1–4–1–4–8–21	ST42/CC5	IB
67060	Wound	Hungary	1–4–8–21	ST42/CC5	1–1–1–4–1–4–8–21	ST42/CC5	IB
4–2–6^a^	Skin	France	1–4–8–6	ST5/CC5	1–1–9–4–1–4–8–6	ST53/CC5	IB
37J+	Skin	Brazil	1–18–8–2	ST57/CC2	1–1–1–18–1–4–8–2	ST57/CC2	IA_2_
29C^a^	Skin	Brazil	17–4–10–12	ST30/CC72	17–4–2–4–4–6–10–12	ST69/CC72	II
28S−a	Skin	Brazil	17–4–10–12	ST30/CC72	17–4–2–4–4–6–10–12	ST69/CC72	II
5–3–4^a^	Acne	France	17–4–10–12	ST30/CC72	17–4–2–4–2–12–10–12	ST72/CC72	II
17A	Skin	Brazil	7–7–12–23	ST75/CC77	7–6–3–7–5–9–12–23	ST75/CC77	III
32P	Skin	Brazil	7–7–12–23	ST75/CC77	7–6–3–7–5–9–12–23	ST75/CC77	III
18S^a^	Skin	Brazil	7–7–12–16	ST76/CC77	7–6–3–7–10–9–12–16	ST76/CC77	III
30F	Skin	Brazil	7–7–12–16	ST76/CC77	7–6–3–7–5–9–12–16	ST77/CC77	III
A9	Acne	Hungary	1–15–2–2	Novel	1–1–1–15–9–13–2–2	Novel (ST92; S)	IA_1_
PF3^a^	Acne	Hungary	1–3–1–1	ST1/CC1	1–1–1–3–8–1–1–1	Novel (ST93; CC1)	IA_1_
PF9	Acne	Hungary	1–10–8–6	Novel	1–1–1–10–1–1–8–6	Novel (ST94; CC4)	IA_1_
PF12	Acne	Hungary	1–9–8–6	Novel	1–1–1–9–9–1–8–6	Novel (ST95; S)	IA_1_
12553	Blood	Hungary	15–4–24–10	Novel	15–4–2–4–2–3–24–10	Novel (ST96; CC6)	II
M34	Wound	Hungary	1–3–25–1	Novel	1–14–1–3–1–1–25–1	Novel (ST97; S)	IA_1_
101320	Surgical sample	Hungary	1–4–8–34	Novel	1–1–1–4–1–4–8–34	Novel (ST98; CC5)	IB
24763	Intraabdominal sample	Hungary	1–4–26–6	Novel	1–1–1–4–1–4–26–6	Novel (ST99; CC5)	IB
63848	Conjunctiva secretion	Hungary	20–4–10–12	Novel	20–4–2–4–2–6–10–12	Novel (ST100; CC72)	II
76793^a^	Oral	Hungary	1–3–1–1	ST1/CC1	1–1–1–3–1–14–1–1	Novel (ST101; CC1)	IA_1_
M8 a	Acne	Hungary	1–3–8–6	ST4/CC4	1–1–1–3–1–14–8–6	Novel (ST102; CC4)	IA_1_
19695^a^	Wound	Hungary	1–3–1–1	ST1/CC1	1–15–1–3–1–1–1–1	Novel (ST103; CC1)	IA_1_
44261	Blood	Hungary	21–3-1–6	Novel	21–1–1–3–1–1–1–6	Novel (ST104; CC1)	IA_1_
56853	Heart implant	Hungary	1–3–1–6	Novel	1–1–1–3–1–1–1–6	Novel (ST105; CC1)	IA_1_
46361	Acne	Hungary	15–19–10–10	Novel	15–4–2–19–2–3–10–10	Novel (ST106; CC6)	II
5–1–3a,b	Acne	France	9–8–14–6	ST85/S	9–1–4–8–6–8–14–6	Novel (ST107; CC107)	IC
2–1–7	Acne	France	17–20–10–12	Novel	17–4–2–20–2–12–10–12	Novel (ST108; CC72)	II
6–2–3^a^	Acne	France	17–4–10–12	ST30/CC72	17–4–2–4–2–15–10–12	Novel (ST109; CC72)	II
8–4–1	Acne	France	1–3–8–2	Novel	1–1–1–3–1–4–8–2	Novel (ST111; CC2)	IA_2_
IT41EA	Acne	UK	1–3–27–1	Novel	1–1–1–3–1–1–27–1	Novel (ST112; CC1)	IA_1_
16J	Skin	Brazil	18–4–20–35	Novel	18–4–2–4–2–6–20–35	Novel (ST113; CC71)	II
11G^a^	Skin	Brazil	7–7–12–16	ST76/CC77	7–6–3–7–5–16–12–16	Novel (ST114; CC77)	III

a ST was not fully resolved or a novel genotype disclosed based on MLST_4_.

b MLST_4_ classified isolate as a singleton, but belongs to the novel CC107 group; phylogroup was, however, correctly identified.

When data for all isolates from this and our previous study was combined (n = 372), the MLST_4_ method resolved a total of 94 STs with a *D* value of 93% (95% CI 91.4–94.6). The full MLST_8_ approach resolved 114 STs with a *D* value of 94% (95% CI 92.4–95.2) (p<0.01). In total, the ST for 91% of all the isolates analysed was correctly determined based on MLST_4_. Our analyses clearly demonstrate, therefore, the potential value of the MLST_4_ method for genotyping of *P. acnes* and classification of isolates into groups with phylogenetic and clinical relevance. Even in the cases where a smaller number of specific STs cannot be completely resolved based on this approach, the data generated will still enable an isolate to be correctly assigned to its phylogroup and CC in almost all cases. It will still be necessary, however, to conduct ‘full’ MLST analysis if novel allelic profiles are generated so the MLST database can be updated or, depending on the epidemiological or phylogenetic/evolutionary questions being addressed, a deeper layer of resolution is required for specific lineages.

### MLST and phylogenetic analysis of uncharacterised HMP *Propionibacterium* species

As part of our ongoing analysis of other propionibacteria that currently comprise the HMP, we examined four uncharacterised *Propionibacterium* isolates currently listed as distinct from other recognized *Propionibacterium* species (http://www.ncbi.nlm.nih.gov/genome/genomes/1140). On the basis of our initial analysis of 16S rDNA sequences, we unexpectedly found that all these isolates appeared to represent strains of *P. acnes* (1485/1486 bp; 99% identity); this was subsequently confirmed based on phylogenetic analysis of *recA* and *rpoB* housekeeping gene sequences (Figure S2). In keeping with their identification as *P. acnes* isolates, it proved possible to characterise the isolates by MLST_8_ analysis. The strain 5U42AFAA was identified as ST1 (type IA_1_; CC1) based on our scheme, and ST29 (type IA_1_; CC18) based on MLST_9_; CC1 and CC18 are synonymous between the two schemes. Isolate 434-HC2 was ST5 (type IB; CC5) based on MLST_8_ and ST36 (type IB; CC36) based on the MLST_9_ approach. Again, both CC5 and CC36 are synonymous complexes between the two typing methods. Both 409-HC1 and CC003-HC2 had the novel genotype ST117 (type IA_1_; CC4) based on MLST_8_ and ST27 (type IA_2_; CC28) by the MLST_9_ method. This difference between the two schemes in respect to their phylogroup classification of both isolates was also previously observed with the isolate HL025PA1, an ST4 (CC4) based on our scheme and ST27 (CC28) based on the Aarhus approach [17]. Comparative genome analysis has shown that the genetic distances between HL025PA1 and CC4 (type IA_1_) or CC28 (MLST_9_, type IA_2_), calculated on the substitution rates of single nucleotide polymorphisms within the core regions, are very similar and not significantly different [24]. Furthermore, genomic interrogation has revealed the presence of genes that are identical or very similar to those present in CC1 (type IA_1_), CC3 (type IA_1_), CC4 (type IA_1_) and CC6 (type II). The difference between the two schemes in their phylogenetic classification of HL025PA1 may reflect the mosaic nature of its genome sequence, and their analysis of completely different sets of genes located in different parts of the genome. A reference document containing MLST data for *P. acnes* strains that form part of the HMP and other whole genome sequencing projects can be found at http://pubmlst.org/pacnes.

### Classification within the type I phylogroup

Type I isolates were originally classified into type IA and IB based on the phylogenetic analysis of *recA* and *tly* genes [10], [11]. More detailed phylogenetic analysis based on MLST_8_ sequence data has, however, revealed that all type IA isolates can be further partitioned into one of two distinct, statistically significant clades, which we have designated types IA_1_ and IA_2_ [17], [26]; these two distinct divisions are further supported at the phylogenomic level [24]. Within the type IA_1_ group, we also see a further subdivision into a number of phylogenetic clusters that essentially correspond to CC1 (clade IA-1 [27]), CC3 (clade IA-2 [27]) and CC4 (clade IB-1 [27]) based on MLST_8_ [17]. Strains within the type IA_2_ clade are also distinct based on biotype and contain fewer non-core genomic regions when compared with type IA_1_ strains, which may result from a lack of rearrangement hot spot family proteins [27]. From a medical microbiology perspective, such classifications are only useful if they are also associated with important traits, such as the expression of specific virulence factors, pathogenicity, antibiotic resistance or association with specific infections or clinical conditions. In the case of type IA_1_ CCs, we see a strong association with moderate-to-serve acne, as described later, while type IA_2_ strains are only recovered at a level similar to that observed with type IB (I-2, Aarhus MLST; clade IB-3 [27]) and II strains, which are generally considered non-acne associated [16], [17], [24]. This clinical observation alone should justify their description as a distinct group from other type IA strains.

While strains within the recently proposed type IC group (which includes HMP strain HL097PA1) form a phylogenetic clade that is highly distinct from all type IA strains [17], [24], [28], they are nonetheless very closely related as evidenced by the expression of dermatan-sulphate binding adhesins, the presence of genomic regions in common with type IA strains, and a strong association with acne [17], [24], [28]. For example, our analysis shows that type IC strains contain a 8.7 kb genomic region found in our previously described type IA_1_ and IA_2_ strains, but not type IB, II or III, that encodes a glycosidase hydrolyse gene, ABC-type peptide uptake operon, and chitinase gene [19]. Despite this, differences from types IA strains do exist at the genomic level, including deleted regions, and all type IC isolates analysed to date do not ferment sorbitol and display resistance to anti-acne antibiotics via mutations in their rDNA genes [17], [28].

### Phylogroups, STs and human infection

**Figure 3:**
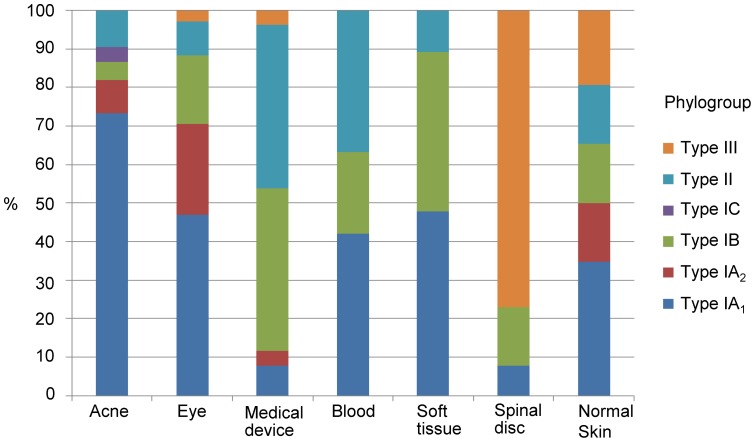
Association of *P. acnes* phylogroups with different clinical conditions and normal skin. Data was obtained from MLST analysis of 372 isolates.

The analysis of a new, large batch of isolates from disparate clinical sources for this study (Table 2) provided the opportunity to further build on our previous work examining the relationship between phylogroups, STs and infection. To facilitate a more robust analysis, data from the isolates used in this (n = 87) and our earlier study (n = 285) were combined (n = 372) (Table S2). The data demonstrates that STs from the *P. acnes* type IA_1_ group (CC1; CC3; CC4) strikingly dominate isolates cultured from acne-affected regions (73%), while those from the type IA_2_, IB, IC, II and III groups are either recovered at much lower rates (<10% per group) or never recovered (type III) (Figure 3) [9], [16], [17], [24]. With isolates recovered from normal facial and upper back skin (n = 97) we find a somewhat more even distribution (p<0.001) (Figure 3). While key STs associated with moderate-to-severe acne (ST1, ST3, ST4) are present in normal skin, they appear particularly enriched in the acne-related samples; particularly ST3 (CC3). For example, ST1 and ST3 alone represent 26% and 14%, respectively of acne isolates, but only 15% and 4%, respectively of isolates from normal skin (Table S2). The ST3 lineage also appears especially associated with rRNA point mutations conferring resistance to tetracyclines, erythromycin and clindamycin [17]. As acneic and normal pilosebaceous follicles will co-exist together on the skin of acne sufferers, it is likely that some type IA_1_ strains isolated from acneic skin will not directly relate to the condition when sampling from follicles [16], [17], [24]. Despite this, the high statistical significance relating type IA_1_ with this common skin condition does provide evidence for a meaningful association. Similar conclusions being reached in different independent studies also provides evidence for this association [16], [17], [24]. For example, Fitz-Gibbon et al. [24] found ribotypes 4/5 and 8, which are synonymous with the MLST_8_ genotypes ST3 (CC3) and ST4 (CC4), respectively are enriched in acne patients, but ribotypes representing type IA_2_ strains show no statistically significant association with the condition. While the apparent capacity of type IA_1_ strains to contribute to the pathophysiology of acne over other phylogroups remains unclear, we can speculate that it may reflect an enhanced ability to adapt to the changing environment created as a normal follicle develops into a lesion. Strains from this division also appear to display increased inflammatory potential and the expression of putative virulence factors, such as dermatan sulphate-binding adhesins (PA25957/PPA2127, DsA1; PA5541/PPA2210, DsA2), which may play important roles in the disease process. The latter cell surface molecules have hypermutable homopolymeric tracts upstream of a putative signal peptide that may give rise to slipped-strand mispairing during replication resulting in either phase or antigenic variation that may be important in immune evasion or adaption to environmental changes [9]. These proteins also have variable numbers of PT repeats towards the mid-region and the carboxyl-terminus that generates differences in molecular mass, as well as the absence of an ATG start codon in type IB strains [9]. Such phase variable adhesins are also found in ‘classical’ pathogenic species, including the SabA adhesin of *Helicobacter pylori* which is implicated in binding to the host gastric epithelium [29]. Epitope mapping studies of DsA1 indicate that the region towards the amino-terminus of this polypeptide is recognized by antibody in serum from acne-positive individuals and contains a CD4^+^ T cell epitope [13]. At the genomic level ST3 strains contain unique regions that are believed to have originated from mobile genetic elements and encode virulence genes [24], while ST4 strains contain a distinct 20 kb island that encodes nonribosomal peptide synthetases which may contribute to increased virulence [27]. With the MLST_8_ scheme we have found that the large majority of isolates from acneic regions of the skin (∼85%) that we previously classified as ST6 (CC6) based on the original Warwick MLST_7_ protocol [9] retain the same ST when revisited with the enhanced resolution of the expanded method (ST1; CC1) confirming our conclusions regarding the clonal nature of this set of isolates. Similarly, reanalysis of type IB isolates originally identified as genotype ST10 (CC10) [9] revealed the same ST (ST5) in nearly all cases (90%) by MLST_8_; type IBs represent a tight phylogenetic cluster with a simple CC structure and strongly predicted founder (ST5; 89% bootstrap value) which indicates a relatively young age. These results therefore validate our previously described conclusions regarding the identification of disseminated clonal lineages, backed up by RAPD analysis [9], and the apparent association of a dominant type IA_1_ epidemic lineage with the skin of acne patients [9]. In addition to acne, we also observed significant numbers of type IA_1_ strains isolated from ophthalmic and other soft tissue infections, as well as blood samples (ure 3), including the common acne-associated lineages ST1 (CC1), ST3 (CC3) and ST4 (CC4); this suggests a broader pathogenic potential. In keeping with this, we have found that the recently completed whole genome sequence of the *P. acnes* isolate C1 [30] (Genbank accession no. CP003877.1), recovered from a Japanese patient with sarcoidosis also represents a type IA_1_ strain belonging to CC3 (new genotype ST115; 1–1–1–3–1–1–22–2) (not included in this study). During this study we also identified a novel type IC genotype, ST107 (Table 3; Table S2); this brings the total number of isolates currently identified for this group to five (three STs). In keeping with previous observations, the ST107 isolate cross-reacted with mAbs primarily specific for types IA (QUBPa1) and II (QUBPa2) [17], [28].

While STs from the type IA_2_, IB, II and III groups do not appear to commonly represent isolates Figfrom acneic regions of the skin, a heightened role in medical device and soft tissue infections is suspected [16], [17], and results from our current MLST isolate collection do lend some support to this view (Figure 3). Of particular note, type II isolates are commonly associated with blood and medical devices, and type IB isolates with medical devices and soft tissue infections; although it is unclear whether such associations are clinically meaningful in many instances. The number of type IA isolates associated with medical devices is low based on our current but large MLST collection; and somewhat in contrast to our previous antibody typing studies in 2005 which found levels almost equalling those of type II strains, with little evidence for type IB, based on a different set of 40 isolates recovered from prosthetic hip joint sonicates [11]. Further studies are required to conclusively determine whether type IB and II lineages truly have an increased potential to colonise and subsequently cause downstream medical device-related infections.

Relevant to our observations on soft tissue infections is a recent study which found that *P. acnes* isolates from cancerous prostate tissue (n = 10) belong to either the type IB (30%) or type II (70%) divisions [31]. This fits with our more recent MLST_8_ publication which similarly found a predominance of type IB (45%; ST5 and ST53) and type II (33%; ST6, ST65 and ST67) genotypes, and to a lesser extent type IA_1_ (22%; ST4 and ST21), amongst a small collection of *P. acnes* isolates recovered from cancerous prostate tissue (n = 9) [17]. Another study on 24 *P. acnes* isolates retrieved from the cancerous prostate tissue of Swedish patients identified the majority as type II (67%), with similar numbers of type IA (21%) and type IB (12.5%) [22]. Combining the typing data from all three independent studies therefore provides support for an association between lineages from the type II phylogroup and cancerous prostates (61% of all isolates; p<0.001). While a possible role for *P. acnes* in prostate cancer development remains controversial, the dominance of STs from this phylogroup over strains from the type I division does provide some evidence to support the view that isolation of *P. acnes* from cancerous prostate samples cannot simply be dismissed as contamination from the skin, at least not in all contexts; this confirms the original seminal observations of Cohen et al. [7]. The observation that type II strains appear capable of causing greater long term infection in a rat model of prostatic inflammation (three months post-infection) when compared with type I strains would therefore appear an important observation when combined with emerging epidemiological data; although bacterial counts were very low [32]. It still remains to be determined, however, whether the presence of *P. acnes* in human prostates may arise from the urethra, especially as type IB and II lineages do appear more common inhabitants of this environment [19], or post-cancer colonisation of hypoxic regions within already growing prostate tumours. Further studies will undoubtedly address these issues. An interesting observation from the analysis of our current isolate database was the relatively high proportion of type III isolates that were associated with spine intervertebral disc material (excised disc protrusion) (Figure 3). We first identified type IIIs as representing a novel *P. acnes* phylogroup in 2008, again mostly in association with spinal disc material [15], which was an interesting observation as *P. acnes* had been described as a possible cause of sciatica [33]. Since then, there has not been any definitive evidence to suggest this lineage is responsible for infection. Indeed, we previously found lineages from this group (ST32, ST75, ST76 and ST77) only in association with healthy skin, tentatively suggesting they may represent ‘true’ commensals [17]; whether such strains with no pathogenic potential do exist has yet to be conclusively established. Proteomic studies with the type III strain 487 have also found that when compared with other phylogroups (IA_1_, IB, & II), it produces fewer detectable secreted proteins after growth in complex media [34]. In the context of their main ‘clinical’ isolation sources to date, primarily spinal disc tissue, and medical devices [15], [17], it will be important to ascertain if this association is real or whether it simply reflects contamination of surgical sites with skin microbiota. This will be important since previous studies have shown that contamination of spinal incision sites during surgery can occur, even after pre-operative skin antisepsis and antimicrobial prophylaxis [35].

### Clonal diversification within *P. acnes*: mutation versus recombination

We have previously shown that the overall *P. acnes* population structure is clonal and in linkage disequilibrium based on index of association (*I_A_*) analyses for our entire isolate collection, as well as unique STs [9], [17]. We also observed a reduction in the *I_A_* value when only STs were considered, which is also in keeping with an epidemic population structure where highly successful clonal lineages emerge and become widely dispersed [9], [17]. This supports observations that particular MLST lineages are especially prevalent and can display intercontinental spread as a result of human migration [16], [17]. A similar epidemic population structure is also seen with the other Gram-positive skin commensal, *Staphylococcus epidermidis* [36]. No evidence of statistically significant recombination was identified in either the housekeeping or putative virulence gene sequences using the phi test (Figure S3), although for a number of the loci (eg., *aroE*, *guaA*, *sodA*) split decomposition graphs did suggest limited recombination events due to the presence of interconnected pathways (Figure S3); again consistent with our previous study which found non-congruence between some housekeeping gene trees [9]. Analysis of concatenated sequences from the population as a whole, however, did reveal levels of recombination that were statistically very significant (phi test p = 0.001).

**Figure 4:**
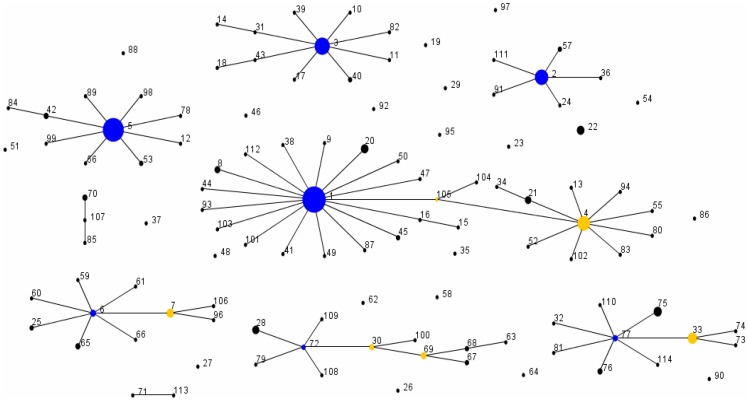
eBURST population snapshot of the current *P. acnes* MLST database. To date, a total of nine clonal complexes, where the isolates share 7/8 loci with at least one other ST in the group, and 21 singletons have been identified. The frequency of each ST within the isolate database is indicated by the size of each circle. Founding genotypes are highlighted in blue and sub-founders in yellow. The previously described separate complexes CC1 and CC4 now form a large single complex with ST1 as the founder (100% bootstrap) and ST4 (98% bootstrap) as a sub-founder; these two clusters are linked via ST105. Note, the spacing between the singletons and clonal complexes is not related to the genetic distance between them.

To estimate the roles that mutation and recombination play in generating clonal diversification within *P. acnes*, the *r/m* parameter was calculated. Such analyses are important if we are to understand how recombination contributes to the evolution of this bacterium, particularly in the context of antibiotic resistance and current discussions around vaccine development. Based on our current database, which is now sufficiently diverse for such an analysis, a total of nine CCs have been identified providing a total of 84 single locus variants (SLVs) for analysis (Figure 4; Table 4). The previously described type IA_1_ CCs, CC1 and CC4 now form two clusters within a large single complex, with ST1 as the founder (100% bootstrap) and ST4 (98% bootstrap) as a sub-founder; these two clusters are linked via ST105 which contains a *recA* gene sequence identical to that from NCTC737 (ST1; CC1) (Figure 4). A total of 39 variants were from the type IA_1_ group, five from type IA_2_, nine form type IB, two from type IC, 20 from type II and nine from type III. A total of 45 SLVs had alleles that differed at only one nucleotide site from the ancestral ST and were not present in any other STs within the database; these SLVs were considered to have arisen by de novo mutation (Table 4). A total of 20 SLVs also had alleles that differed at only one nucleotide site from the ancestral ST, but these alleles were also present in other STs within the database. Of these, seven were still considered to have arisen by mutation as their presence in other STs clearly appeared to reflect vertical descent based on eBURST analysis, while the remainder were classified as recombination events (Table 4). The remaining 19 SLVs all had more than one nucleotide difference consistent with recombination. For the population has a whole, the per-allele *r/m* parameter was calculated as 1∶1.6 and the per-site *r/m* parameter as 3∶1 using this current dataset. This demonstrates that for *P. acnes*, alleles are marginally more likely to change by mutation (∼1.5-fold) but individual nucleotide sites by recombination. While rates of recombination within *P. acnes* are less than that found in many other bacterial species, levels are still sufficiently high to ensure an important role in the evolution of the bacterium, and this plasticity in the gene pool has been further evidenced by the analysis of whole genome sequences which reveals mosaics within the population [37].

**Table 4:** Roles of mutation and recombination events in the generation of SLV alleles within *P. acnes.*

Phylogroup	ST of clonal ancestor	ST of SLV	Variant locus	Ancestral allele no.	SLV allele no.	No. of nucleotide differences^a^	Other STs with SLV allele^b^	Genetic event^c^
IA_1_	1	8	*tly*	1	3	1 (A→G)	46S	R
IA_1_	1	9	*tly*	1	4	1 (G→A)	None	M
IA_1_	1	16	*gmk*	1	10	1 (C→G)	15 (CC1)	M^d^
IA_1_	1	20	*aroE*	1	5	1 (C→T)	None	M
IA_1_	1	38	*tly*	1	16	1 (A→C)	None	M
IA_1_	1	41	*guaA*	3	6	1 (C→T)	None	M
IA_1_	1	44	*aroE*	1	11	1 (G→A)	None	M
IA_1_	1	45	*gmk*	1	6	1 (G→A)	None	M
IA_1_	1	47	*aroE*	1	4	1 (C→T)	None	M
IA_1_	1	49	*guaA*	3	9	1 (G→A)	95S	R
IA_1_	1	50	*aroE*	1	12	2 (C→T; T→C)	None	R
IA_1_	1	87	*gmk*	1	11	1 (G→A)	None	M
IA_1_	1	93	*lepA*	1	8	1 (C→T)	52 (CC4)	R
IA_1_	1	101	*sodA*	1	14	1 (C→T)	14 (CC3);102 (CC4)	R
IA_1_	1	103	*atpD*	1	15	1 (C→T)	None	M
IA_1_	1	105	*camp2*	1	6	1 (T→G)	17x STs (CC1,4,5,107,S)	R
IA_1_	1	112	*tly*	1	27	1 (T→C)	None	M
IA_1_	3	10	*camp2*	2	4	1 (C→A)	None	M
IA_1_	3	11	*tly*	2	5	2 (C→A; A→C)	None	R
IA_1_	3	17	*atpD*	1	10	1 (C→T)	None	M
IA_1_	3	31	*camp2*	2	3	1 (A→G)	14 (CC3)	M^d^
IA_1_	3	39	*camp2*	2	20	6 (n/s)	None	R
IA_1_	3	40	*atpD*	1	5	1 (C→T)	None	M
IA_1_	3	43	*aroE*	1	8	1 (C→T)	18 (CC3)	M^d^
IA_1_	3	82	*tly*	2	21	3 (n/s)	None	R
IA_1_	4	13	*tly*	8	7	1 (A→C)	None	M
IA_1_	4	21	*guaA*	3	1	1 (A→G)	34 (CC4)	M^d^
IA_1_	4	52	*lepA*	1	8	1 (C→T)	93 (CC1)	R
IA_1_	4	55	*atpD*	1	12	1 (G→A)	None	M
IA_1_	4	80	*atpD*	1	11	1 (G→A)	None	M
IA_1_	4	83	*camp2*	6	30	2 (G→A; G→T)	None	R
IA_1_	4	94	*guaA*	3	10	19 (n/s)	62S	R
IA_1_	4	102	*sodA*	1	14	1 (C→T)	14 (CC3); 101 (CC1)	R
IA_1_	16	15	*tly*	1	6	5 (n/s)	None	R
IA_1_	21	34	*camp2*	6	22	1 (C→T)	None	M
IA_1_	31	14	*sodA*	1	14	1 (C→T)	101 (CC1);102 (CC4)	R
IA_1_	43	18	*camp2*	2	5	1 (A→G)	29S	R
IA_1_	105	4	*tly*	1	8	2 (A→C; A→G)	27x STs (CC2,4,5,S)	R
IA_1_	105	104	*aroE*	1	21	1 (A→G)	None	M
IA_2_	2	24	*camp2*	2	9	1 (G→A)	None	M
IA_2_	2	36	*guaA*	5	13	1 (C→T)	None	M
IA_2_	2	57	*guaA*	5	18	1 (G→T)	None	M
IA_2_	2	91	*tly*	8	9	1 (T→G)	None	M
IA_2_	2	111	*guaA*	5	3	1 (T→G)	42x STs (CC1,2,3,4,S)	R
IB	5	12	*camp2*	6	31	1 (A→C)	None	M
IB	5	42	*camp2*	6	21	1 (C→G)	84 (CC5)	M^d^
IB	5	53	*gmk*	1	9	2 (2x T→C)	51S	R
IB	5	56	*gmk*	1	12	1 (G→A)	None	M
IB	5	78	*camp2*	6	29	1 (C→A)	None	M
IB	5	89	*camp2*	6	1	1 (G→T)	22x STs (CC1,5,S)	R
IB	5	98	*camp2*	6	34	1 (T→C)	None	M
IB	5	99	*tly*	8	26	1 (G→A)	None	M
IB	42	84	*aroE*	1	19	1 (C→T)	None	M
IC	107	70	*camp2*	6	14	1 (T→G)	None	M
IC	107	85	*gmk*	4	5	1 (T→C)	None	M
II	6	7	*aroE*	17	15	1 (G→A)	96 (CC6);106 (CC6)	M^d^
II	6	25	*atpD*	4	9	1 (T→G)	None	M
II	6	59	*aroE*	17	13	17 (n/s)	None	R
II	6	60	*aroE*	17	3	18 (n/s)	62S	R
II	6	61	*aroE*	17	1	19 (n/s)	63x STs (CC1,2,3,4,5,S)	R
II	6	65	*camp2*	10	19	1 (C→T)	None	M
II	6	66	*camp2*	10	11	4 (n/s)	26S	R
II	7	96	*tly*	10	24	1 (G→A)	None	M
II	7	106	*guaA*	4	19	1 (T→C)	None	M
II	30	69	*lepA*	2	4	1 (A→G)	62,63,64,67,68 (CC72,S)	R
II	30	100	*aroE*	17	20	1 (C→T)	None	M
II	68	63	*aroE*	17	14	13 (n/s)	None	R
II	69	67	*camp2*	12	17	3 (n/s)	None	R
II	69	68	*camp2*	12	18	2 (T→G; C→T)	62,63 (CC72;S)	R
II	71	113	*camp2*	12	35	1 (C→T)	None	M
II	72	28	*guaA*	4	16	1 (C→T)	None	M
II	72	30	*sodA*	12	6	1 (C→T)	9x STs (CC72,S)	R
II	72	79	*camp2*	12	28	1 (A→G)	None	M
II	72	108	*guaA*	4	20	1 (C→T)	None	M
II	72	109	*sodA*	12	15	1 (G→A)	None	M
III	33	73	*guaA*	7	11	4 (n/s)	None	R
III	33	74	*camp2*	16	27	1 (C→T)	None	M
III	77	32	*camp2*	16	15	2 (C→T; G→A)	None	R
III	77	33	*tly*	12	13	1 (T→C)	73 (CC77);74 (CC77)	M^d^
III	77	75	*camp2*	16	23	1 (G→A)	None	M
III	77	76	*lepA*	5	10	1 (C→G)	None	M
III	77	81	*gmk*	3	7	1 (C→A)	None	M
III	77	110	*camp2*	16	32	2 (G→A; T→C)	S90	R
III	77	114	*sodA*	9	16	1 (C→T)	None	M

a n/s =  not shown (number of base changes ≥3).

b S = singleton.

c R = recombination; m = mutation.

d Classified as mutation due to clear evidence of vertical descent only.

### Intra-phylogroup recombination

While *P. acnes* is in linkage disequilibrium overall, we previously observed that the association between alleles appears less significant within the main phylogroups [17]. This suggested that rates of horizontal gene transfer may differ throughout the population. With our increased MLST data set, we explored this possibility in more depth by examining *I_A_* values within types IA, IB, II and III. Within the type I clade (IA_1_, IA_2_, IB, IC), *I_A_* values were still consistent with linkage disequilibrium when all isolates and STs were analysed (p<0.001) (Table 5), but a consistent reduction in *I_A_* values was observed indicating that the association between alleles was less significant. A similar pattern was observed when isolates and STs from the IA cluster (IA_1_, IA_2_) were just considered (Table 5). In both cases, levels of recombination within the groups were significant by the phi test (Table 5), and evidence of reticulation indicative of recombination events within the type IA group also evident upon splits graph analysis (Figure 2). With the type II division population, linkage disequilibrium was detected when all isolates were considered, although the *I_A_* value was low (*I_A_* 0.0485), but linkage equilibrium was identified when only STs from our updated dataset were analysed (*I_A_* −0.0201; p>0.1). While levels of recombination were non-significant by the phi test, this was marginal and clear evidence of potential recombination events within the type II cluster were observed within the splits graph (Figure 2). Similarly, with the type IB phylogroup, linkage disequilibrium was detected with isolates and linkage equilibrium with just STs (Table 5). This difference likely reflected the dominant presence of ST5 (CC5) within this population producing a dilution in the observed effect of recombination. There was, however, no statistically significant measurement of recombination by the phi test. With the type III phylogroup we observed linkage equilibrium when both isolates and STs were considered, but no evidence of recombination was detected by the phi test (Table 5). With both the type IB and III populations, however, sample sizes were smaller and this could affect the statistical robustness of the linkage analysis in these cases [38].

**Table 5:** *I_A_* values for isolates and STs from the whole *P. acnes* population and major phylogroups (IA, IB, II & III).

		*p* value		
Collection^a^	*I_A_* value	Parametric	Simulation	phi test
**All**				
Isolates (n = 372)	0.4847	<0.001	<0.01	–
STs (n = 114)	0.3778	<0.001	<0.01	0.001
**Type I**				
Isolates (n = 288)	0.1960	<0.001	<0.01	–
STs (n = 75)	0.1180	<0.001	<0.01	0.001
**Type IA**				
Isolates (n = 216)	0.1412	<0.001	<0.01	–
STs (n = 61)	0.0437	<0.001	0.03	0.005
**Type IB**				
Isolates (n = 67)	0.0548	<0.001	<0.01	–
STs (n = 11)	–0.0204	1.000	0.69	1.000
**Type II**				
Isolates (n = 53)	0.0485	<0.001	<0.01	–
STs (n = 28)	0.0201	0.187	0.150	0.096
**Type III**				
Isolates (n = 31)	–0.0546	1.000	0.920	–
STs (n = 11)	–0.0021	1.000	0.630	1.000

a Type IC not included as a separate group as sample size too small.

The observation of heightened rates of horizontal gene transfer (HGT) within, but not between, the large type I and II phylogroups may reflect the very recent observation that type II lineages contain clustered regularly interspaced short palindromic repeats (CRISPR)/Cas loci which confer immunity to bacteriophages and foreign mobile genetic elements [27], [39]. Sequence analysis of CRISPR spacers in type II strains has found evidence of previous challenges from *P. acnes*-specific phages and two mobile genetic elements that are found almost exclusively in type I *P. acnes* strains [39]. The CRISPR/Cas system may therefore restrict the acquisition of horizontally transferred DNA from the type I division; although some sharing of alleles between the type I and II clusters was observed (Table 4). Reduced rates of horizontal gene transfer between types I, II and III are also likely to reflect ecological differences, with recombination being much more likely between members of the same habitat. We can speculate that adaptation to specific ecological niches within the human host (Ecotype model) [40], as opposed to genetic drift, has been the primary driver behind the emergence of the major lineages of *P. acnes*. This may also explain, at least in part, the differences in opportunistic infection profile.

### Selective pressure on ‘putative’ virulence loci

All housekeeping gene loci had *d_N_/d_S_* ratios consistent with stabilising or negative selection (<1.0). Furthermore, Tajima's *D* values did not deviate significantly from zero (p>0.1) consistent with a null hypothesis of mutation-drift (neutral) evolution (Table 1). In keeping with our earlier observation that the putative virulence genes *tly* and *camp*2 had levels of genetic diversity similar to the housekeeping loci, we also observed *d_N_/d_S_* ratios <1.0 (Table 1) and values for Tajima *D* (Table 1), as well as Fu and Li's test of selection (data not shown), that were not significantly different from zero whether using distinct allele sequences or those from the complete isolate collection. This is in line with our previous investigations which demonstrated that these genes displayed no clear evidence of diversifying selection by SLAC (p<0.1 threshold), MEME (p<0.05 threshold) or PARRIS (p<0.1 threshold) analyses using recommended cut-off values [17].

In this study, we scrutinised the *tly* and *camp*2 gene sequences in more detail using alternate FEL and REL tests of selection [41]. Under the FEL model (p<0.1 threshold), which is especially valuable with intermediate-to-large datasets, no evidence of sites under diversifying selection were identified for either gene using both REV and HKY85 models of substitution. For *camp2*, however, a total of 15 sites were identified as being under negative selection with the HKY85 model of substitution and 18 sites using REV; these reduced to five and seven sites, respectively when a threshold of p<0.05 was set. With the *tly* gene, six sites were identified as under negative selection with HKY85 and four sites with Rev; these both reduced to zero at p<0.05. With the REL model (Bayes factor threshold of 50), which is useful for small or low divergence alignments, no sites were identified as being under diversifying selection for *camp2* using both HKY85 and REV, but 33 were highlighted as being under negative selection. These results did not change at a Bayes factor threshold of 100. For the *tly* gene, no negative sites of selection were identified using either HKY85 or REV models. Three sites were identified as being under potential diversifying selection with REV; this reduced to zero at a Bayes factor threshold of 100. With HKY85, no diversifying or negative sites of selection were identified. These analyses, combined with other tests of selection, provide little evidence for any positive selection in the history of these putative virulence genes. Although REL highlighted three marginally significant *tly* sites that may be under diversifying selection using the REV model of substitution, the results were inconsistent with the many other tests of selection. As REL is susceptible to false positive results, the biological significance of these sites remains uncertain. Overall, the results are concordant with the view that these putative virulence loci have co-evolved alongside housekeeping genes [16], [17]. This helps explain why previous analyses with *tly* and *camp2* gene sequences generate phylogenetic trees that are essentially congruent with those based on housekeeping loci [10], [11].

### CAMP factor 2 and Tly: more than just virulence proteins?

*P. acnes* is unusual in that its genome codes for an expanded family of five CAMP factor homologues, with putative N-terminal signal peptide cleavage sites, which appear to be expressed at different levels between phylogroups [10]. Amino acid sequence identity between these homologues varies from approximately 35% to 50%. Although the function of these putative virulence proteins in *P. acnes* has not been clearly resolved, *in vitro* studies provide some evidence that CAMP2 may be an important co-haemolytic factor [42], [43]. Previous *in silico* analysis has also demonstrated some sequence identity between the Fc-binding region of *Staphylococcus aureus* protein A and *P. acnes* CAMP factors 1 and 3 suggesting they may have immunoglobulin-binding capabilities [10]. Other studies have shown the protein to display cytotoxic properties against keratinocyte (HaCaT) and macrophage (RAW264.7) cell lines, and to stimulate skin inflammation in mouse ears [43]. Passive immunization of mice with anti-CAMP factor serum abrogates *P. acnes*–induced inflammation in this model; although this is clearly not an appropriate system to mimic the unique and harsh environment found within an inflammatory acne lesion. These cytotoxic effects may reflect, in part, interactions between CAMP2 and host cell acid sphingomyelinase [43]. In contrast, studies with a *camp2* knock-out gene mutant did not find any significant alteration in the transcriptomic response of HaCaT keratinocyte cells to *P. acnes*, but *camp 4* knockout mutants did cause the deregulation of 29 genes, including sphingomyelinase 3 [42]. The suggested pathogenic role of *camp2* has led to its proposal as a potential therapeutic target for the treatment of acne and other *P. acnes*-related diseases via passive immunoprotection with human monoclonal antibodies or vaccination [43], [44]. Putative targets for immunotherapy should ideally contain no evidence of any positive selection; such pressures may lead to the generation of variable epitopes that compromise future clinical use [45]. The demonstration that *camp2* gene sequences display no clear evidence for episodic or pervasive positive selection using a broad range of selection tests, and appear to experience a very modest rate of recombination (phi test p = 0.727), may be important observations in the context of its proposal as a cell surface immunotarget. The *r/m* parameter values calculated for *camp2* (1∶1.4 per allele; 1.7∶1 per site) and also *tly* (1∶1.6 per allele; 1.6∶1 per site) fall within the range of values determined for the housekeeping loci, further arguing for their co-evolution with the core genome [17]. The observation that *camp2* and *tly* experience evolutionary forces more in keeping with housekeeping genes is in contrast to that often observed with cell surface-associated proteins from other bacterial species, where increased diversity, mosaic allele structures and evidence of positive selection are present [46]. In the case of *P. acnes,* this may simply reflect a lack of immunogenic pressure within the cutaneous environment; future more detailed analysis of the different selective pressures acting on other secreted/cell surface-associated proteins of *P. acnes* may help establish this possibility. Co-evolution with the core genome may also provide some evidence that these putative virulence genes play important roles in the commensal/normal existence of *P. acnes*, as well as a pathogenic lifestyle, thus constraining their functional diversity and preventing their loss from the population [16], [17]. The apparent detection of purifying selection upon analysis of the *camp2* gene using FEL and REL tests provides some support for this alternate view, at least for this locus; with *tly* we did also find some suggestion of negative selection with the REL test, but this was at best limited and only marginally significant. In relation to CAMP factor 2, previous immuno-detection and proteomic studies have observed the production of this protein in all strains examined [10], [34]. These include strains from the type II and III phylogroups, which appear to contain lineages which may be ‘true’ commensals. There is a growing recognition that the term ‘virulence factor’ may need to be redefined, and when such determinants are also widely disseminated in commensal strains the term ‘niche factor’ or ‘host adaptation factor’ may be more appropriate to reflect their potential importance in colonisation and survival within the host [47]. Even local damage to host cells in the surrounding milieu of a bacterial cell may prove beneficial in this context, providing particular micronutrients for survival and persistence [47]. Examples of potential ‘niche factors’ include BilE for gastrointestinal survival in *Listeria* and Tad IV pili in *Bifidobacterium breve* [48], [49]. Also, an example of a virulence factor with an important dual function is the PEB1a protein of *Campylobacter jejuni*, a cell surface antigen with a major role in adherence and host colonisation, but also a periplasmic binding protein component of an aspartate/glutamate ABC transporter essential for optimal microaerophilic growth [50]. It is important to remember that skin commensals also have to compete with other microbiota for survival, growth and immune evasion. On this basis, the mechanisms adopted to achieve these lifestyle challenges are likely to overlap somewhat with those utilised by pathogenic strains [47]. Of particular relevance to this study are recent investigations which suggest a role for Tly other than just a hypothetical haemolysin/ cytotoxin in *Mycobacterium tuberculosis*, a member of the order *Actinomycetales* to which *P. acnes* also belongs [51], [52]. It has been proposed that the Tly protein family needs re-annotating and redefining as an RNA-binding FtsJ-like methyltransferase involved in ribosomal biogenesis [52]; the *M. tuberculosis* Tly lacks a signal peptide and transmembrane helices consistent with cytoplasmic localization [52]. We have similarly found no evidence for a signal sequence cleavage site within P. acnes Tly proteins (data not shown). While it is unclear how this work relates to the Tly protein of *P. acnes*, which is 50% identical to the *M. tuberculosis* Tly sequence, it could provide one explanation for the absence of clear diversifying selection within the gene which actually functions as a housekeeping methyltransferase, possibly alongside a role as a haemolysin [51].

### CAMP factor homologues in other human propionibacteria

The evolutionary history of CAMP factor homologues in *P. acnes* has not been addressed; and there is no evidence for their presence in the genome sequences of the non-cutaneous ‘dairy’ or ‘classical’ group species *Propionibacterium freudenreichii subsp. shermanii* (CIRM-BIA1; GenBank accession no. FN806773) or *Propionibacterium acidipropionici* (ATCC4875; GenBank accession no. CP003493), suggesting a potential role in human colonisation [53], [54]. To investigate whether CAMP homologues are present in other human propionibacteria, we interrogated the available whole genome sequences of *Propionibacterium propionicum* (F0230a, GenBank accession no. CP002734), the newly described sister taxon of *P. acnes*, *Propionibacterium humerusii* (P08, GenBank accession no. NZ_AFAM00000000; HL037PA2, GenBank accession no. ADYH00000000; HL037PA3, GenBank accession no. ADXV00000000 and HL044PA1, GenBank accession no. ADZU00000000) [55], and *Propionibacterium avidum* (ATCC25577, GenBank accession no. AGBA00000000; 44067, GenBank accession no. CP005287 [56]).

**Figure 5:**
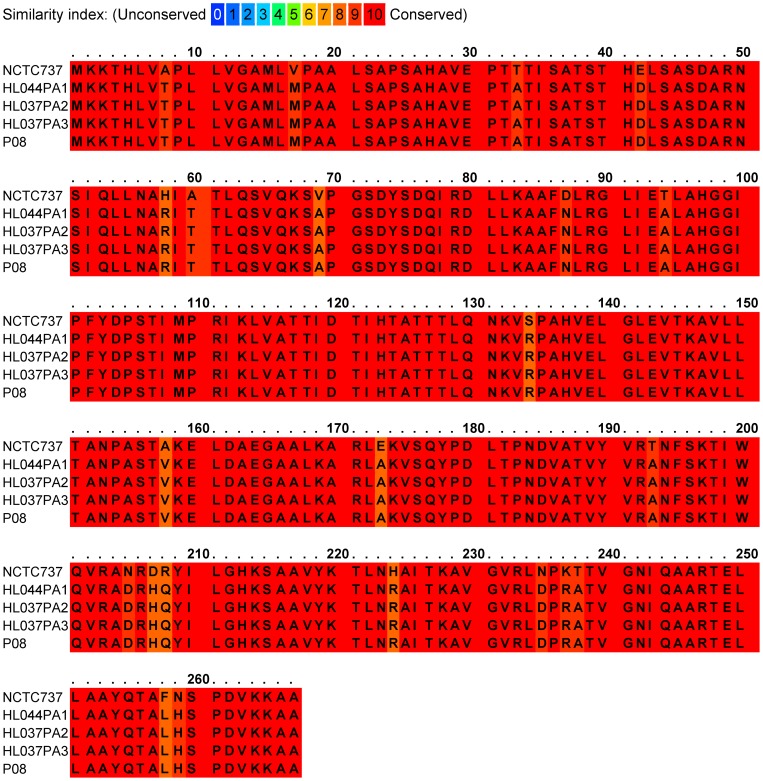
Alignment of CAMP factor 2 amino acid sequences from *P. acnes* type strain NCTC737 (type IA_1_; ST1; CC1) and *P. humerusii* strains HL044PA1, HL037PA2, HL037PA3 and P08. Overall identity between the homologues from these two species based on their protein sequence is 92%. The N-terminal putative signal sequence cleavage site occurs at the AHA-VE sequence (start residue position, 26).

Although our *in silico* analysis provided no clear evidence of any CAMP factor genes in *P. propionicum*, a species responsible for actinomycosis and ondodontic infections [57], nucleotide sequences representing all five homologues were identified in the genome of *P. humerusii* strains. Intact *camp2*, *camp3* and *camp5* gene sequences were present in all four strains, and the resulting amino acid sequences had high identities, ranging from 84–98%, when compared to their equivalent homologues in the *P. acnes* type strain NCTC737 (type IA1 lineage; ST1) which we chose for comparison (Figure 5; Figure S4). In particular, the CAMP3 protein displayed a very high identity with its *P. acnes* counterpart (98%). The *CAMP1* protein in the HMP strains HL037PA2, HL037PA3 and HL044PA1 also had a high level of amino acid identity (91%) to the CAMP1 homologue in P. acnes (Figure S4), but strain P08 had a camp1 gene sequence that gave rise to a truncated protein product (data not shown). The genome from strain P08 is still at the draft assembly stage and it is, therefore, unclear if this reflects a true result or a currently uncorrected sequence error. The *camp4* gene, while present in all four strains, appears to give rise to a truncated product that is ∼55% of the length of the equivalent homologue in NCTC737; this appears to have arisen via the introduction of a stop codon. No clear evidence for CAMP factor homologues 1, 2 and 4 was found in the genomes of *P. avidum*, but *camp3* and *camp5* genes were present with amino acid identities of 92–93% and 80%, respectively when compared to the equivalent homologues in both *P. acnes* and *P. humerusii* (Figure S4). We also examined the presence of the CAMP factor homologues in the *Propionibacterium* isolate SK182B-JCVI, which is currently being sequenced as part of the HMP. While the 16S rDNA sequence of this isolate shares approximately 99% identity with the sequence from *P. acnes* NCTC737, it is clearly a distinct taxon from all other *P. acnes* strains when more rapidly evolving protein-encoding housekeeping loci, such as *recA* (91% identity) and particularly *rpoB* (89–91% identity), are considered (Figure S2); within P. acnes, the main genetic divisions (types I, II and III) are 98–99% identical based on both *recA* and *rpoB*. Further analyses will be required to determine the exact relationship of this strain to *P. acnes* and other propionibacteria. Within this novel taxon, four CAMP factors were identified; three of the amino acid sequences matched CAMP factor 2 (80% identity), CAMP factor 3 (99% identity) and CAMP factor 4 (91% identity) when compared to their equivalent homologues in *P. acnes* NCTC737. The fourth sequence had 65% identity to CAMP factor 1 and 55% identity to CAMP factor 5. The relationship between CAMP factor homologues and propionibacteria based on available genome data is illustrated in Figure S5.

The generation of protein families in prokaryotes, as in eukaryotes, has been assumed to result primarily from intragenomic gene duplication events generating paralogue gene sequences [58]; genome duplications in organisms can result from ancestral whole genome duplications, followed by massive gene loss and specialization, or by duplication of a single gene or group of genes [59]. One very recent study has, however, challenged this view and suggested that expansion of gene families in bacteria via HGT and incomplete xenologous gene replacement may be much more important than first thought [60]. While duplicated sequences are often co-localized as tandem repeats, genome rearrangements/plasticity may complicate this picture resulting in the dispersal of paralogs throughout the chromosome; in contrast, xenologs should be randomly distributed as a result of HGT [60]. In the case of the CAMP factor gene family, *camps* 1, 4 and 5 lie within a relatively limited region of the whole *P. acnes* genome (type IB isolate 6609; positions 1305067–1462007), while *camp* 2 (position 756691–757494) and 3 (position 2282724–2283539) are more widely dispersed. In regards to gene duplication, it provides an opportunity for a rapid period of genetic change due to relaxed selective constraints, and represents a significant mechanism for the emergence of genes with the same, altered or novel cellular functions via mutation, drift and selective pressures [61]. Based on our current understanding, these include ecoparalogs, which are functionally equivalent genes adapted to perform under different ecological conditions [62], subfunctionalization, where the ancestral functions are partitioned between paralogue sequences [63] and, less frequently, neofunctionalization, which generates a new function in one of the duplicates which is subsequently maintained by purifying selection [64]. In contrast, because xenologs are related but distinct proteins they can generate an advantageous new function immediately upon integration to the genome, thus providing a potent method of innovation and long-term adaptive expansion of protein families [60].

On the basis of previously described co-localization and sequence similarity criteria [60], and the assumption that genomic rearrangements occur at a low rate, it would appear that HGT has played some role in the expansion of the CAMP factor family. Irrespective of the exact genetic mechanism(s) and series of events underlying expansion of the CAMP factor protein family, we can be certain that evolution of this family was a pre-speciation event. The observation of similar genomic locations amongst equivalent CAMP homologues in different propionibacteria provides evidence to support this view (Figure S6). On the basis of a gene duplication model, CAMP factor homologues would, therefore, represent ‘out-paralogs’ (alloparalogs). Using the data currently available from whole genome sequencing projects we can now propose a hypothetical scenario for CAMP factor evolution in propionibacteria via expansion of this protein family in a lineage that gave rise to a common ancestor of *P. acnes, P. humerusii, P. avidum and P. propionicum*. While the exact biological role played by each CAMP factor still remains to be determined, the distinct amino acid identities observed between members of the family may highlight possible functional differences. The presence in *P. humerusii* of four CAMP homologues, with high amino acid identity to those in *P. acnes*, again highlights the close taxonomic relationship between these species, and indicates that the respective proteins may perform similar functions in both taxa; the presence of a stop codon in the *camp4* sequence of *P. humerusii* suggests this protein is no longer expressed in an active form in this species. The absence of CAMP factors 1, 2 and 4 in *P. avidum* and all CAMP sequences in *P. propionicum*, the closely related sister taxon of *P. avidum*, leads us to tentatively suggest that in these descendant species their presence has not proven beneficial and, consequently, they have been deleted from the genome over time; gene loss due to a lack of fitness is a common feature of the evolutionary process, although xenologs appear to persist longer than paralogs possibly as a result of greater adaptive roles [60]. This process may also occur to remove genes whose function interferes with the invasion of a new niche [65]. These observations are consistent with the idea that in *P. humerusii* and *P. acnes,* *camp* gene sequences may encode proteins that have some degree of functional diversity, and provide a selective advantage (eg., metabolically and /or ecologically) for their existence within the human host as niche/ host adaptation factors, in addition to their role in as putative virulence factors, hence their retention within these genomes. Circumstantial evidence that supports such a possibility has come from immunofluorescence studies with a monoclonal antibody to CAMP factor 1, which has shown expression of the protein amongst both follicular and epidermal *P. acnes* populations in healthy/normal skin [66]. Interestingly, *P. humerusii*, like *P. acnes*, has been found in the extreme and stressful environment of the pilosebaceous follicle providing evidence that it may have the capacity to survive within similar environmental niches [24]. Although the original strain was isolated from the humeral membrane of a patient who underwent revision of a failed total shoulder arthroplasty, the ability of this species to cause opportunistic infections has yet to be firmly established [55]. In contrast, water and substrate availability appear dominant forces in the ecology of *P. avidum* which preferentially colonises moister areas of the skin, such as the vestibule of the nose, axilla and perineum; *P. propionicum* is primarily isolated from the oral cavity, although it may not actually represent part of the normal human microbiota [67]. While previous studies based on the analysis of *P. acnes* grown on anaerobic blood agar plates and brain heart infusion broth have demonstrated CAMP factor expression [10], [34], such data for homologues in *P. humerusii* and *P. avidum* are currently not available; such studies will be essential if we are to further investigate a possible role in commensal existence. For almost all of the CAMP factor protein sequences examined from *P. humerusii*, *P. avidum* and SK182B-JCVI, the characteristic -AXA- N-terminal putative signal sequence cleavage site is present, with the exception of the *P humerusii* CAMP 1 factor where a -VHA- motif replaces -AHA- in P. acnes (Figure S4). Future work will hopefully determine the exact functions of the different *camp* genes in *P. acnes* and whether their retention within the genome does indeed reflect adaption to changing environments. This information, combined with emerging genome data for other *Propionibacterium* species, will enable us to further explore and discuss different aspects of this possible CAMP factor evolution scenario and, if necessary, refine or change the hypothesis in light of new data.

### CAMP factor 2 protein as a therapeutic target?

If CAMP2 and indeed other CAMP factor homologues do play an important role in the survival of *P. acnes* within the human host, as well as contributing to a pathogenic lifestyle, then we could speculate that selecting CAMP2 as a therapeutic target may potentially impact on those lineages associated with maintaining health, in addition to those with a heightened capacity to cause disease. The potential relevance of this issue has been highlighted by a study demonstrating that fermentation of glycerol by *P. acnes* can function as a skin probiotic for growth suppression of community-acquired methicillin-resistant *Staphylococcus aureus* [68]. Unintended targeting of commensal lineages may, therefore, impact on the skin's ability to prevent colonisation and infection by more pathogenic organisms. This is likely to prove more of an issue for CAMP factor-targeted vaccines, rather than local targeting of secreted CAMP factor in acne lesions via topical passive immunisation, which has also been suggested as a treatment via transdermal delivery with microneedles [44]. Passive neutralization of CAMP factor in infected lesions appears, as we might expect, not to influence the colonization of the bacterium at other body sites [44]; although it should be noted that these studies were only performed in a mouse ear model of skin inflammation and bacteria were only enumerated after an overnight incubation. In the future, it will be important to determine exactly what effect, if any, a possible CAMP-factor targeted vaccine would have on ‘commensal’ strains. This will be essential as no definite evidence currently exists that CAMP factor 2 plays any significant or central role in the pathophysiology of acne vulgaris.

### Analysis of genome synteny between *P. acnes*, *P. humerusii* and *P. avidum*

**Figure 6:**
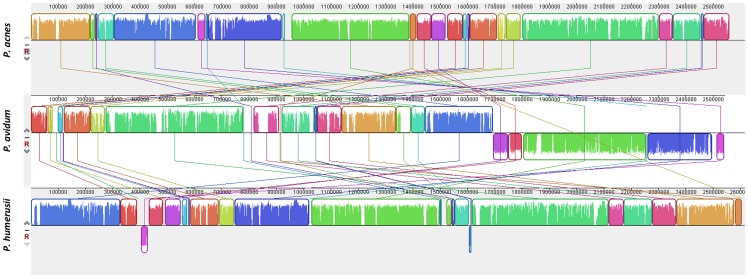
MAUVE visualisation of synteny between *P. acnes*, *P. avidum* and *P. humerusii* genomes. Pairwise alignments of genomes were generated using the progressive MAUVE algorithm (v2.3.1). The sequence similarity in the pairwise alignment of *P. acnes* (6609, type IB ST5, CC5) and *P. avidum* (ATCC25577) was 53.2%. The similarity between *P. acnes* (6609) and *P. humerusii* (P08) was 69.3%, and between *P. avidum* (ATCC25577) and *P. humerusii* (P08) 52.7%.

The high amino acid sequence identities between shared CAMP factor homologues in the genomes of *P. acnes*, *P. avidum* and *P. humerusii* prompted us to investigate the relationship among the complete genome sequences using progressiveMauve, which is a multiple genome alignment tool [69]. We found that *P. acnes* shows a relatively high synteny with *P. humerusii*, differing by 30.7% in the pairwise alignment (pairwise genome content distance) and with only two inverted local collinear blocks (LCB) (Figure 6). In contrast, *P. avidum* has lower synteny with both *P. acnes* and *P. humerusii*, differing by 46.8% and 47.3%, respectively, and with five inverted LCBs. These results therefore mirror the phylogenetic relationships between the species as based on 16S rDNA analysis (Figure S5), and further illustrate the close evolutionary relationship that exists between *P. acnes and P. humerusii*.

## Conclusions

*P. acnes* has been an underestimated and underreported cause of different human infections and maladies [70]. The demonstration via phylogenetic and genetic population studies that particular genetic divisions and lineages exist with a heightened ability to cause disease, while others appear health associated, now provides a solid platform for evidential-based, hypothesis-driven, research into the exact role of this organism in human disease. Against this background, molecular characterisation of isolates by MLST prior to further analyses, such as antimicrobial susceptibility testing or host-pathogen interactions, will now provide a valuable foundation on which we can build our understanding of the relationship between specific lineages and pathogenicity. This will be vitally important as we seek to develop new and specific therapeutic and diagnostic strategies for *P. acnes*-related diseases. MLST represents an ideal approach for isolate characterisation due to the highly portable nature of the methodology and the non-subjective interpretation of the results, unlike gel-based typing systems [71]. The demonstration in this study that MLST analysis based on only four loci, along with simple cross reference to allelic profiles available in the *P. acnes* MLST database (http://pubmlst.org/pacnes/), provides accurate phylogenetic data for researchers, but with a significant reduction in costs, labour and time, should prove beneficial for researchers within this context.

While acne is not a life threatening condition, it can have severe psychological effects on sufferers. To date, the mainstay treatment for this skin condition has been the administration of topical retinoids and topical and oral antibiotics [72]. In recalcitrant cases isotretinoin may be prescribed, but this drug can have severe side effects. The development of a vaccine against acne has long been sought after, and its possible effect on other *P. acnes*-related diseases, particularly prostate cancer (if a causal link can be proven), may prove an added bonus. Current discussions centred on the use of *P. acnes* CAMP2 as a therapeutic target for acne are interesting, and while *in vitro* and mouse ear models of infection provide evidence of a potentially efficacious outcome upon targeting of this protein, this must be balanced against the fact that the role of CAMP2 in the pathophysiology of acne has not been established. In addition, current animal models used to study the potential role of this ‘virulence’ factor in acne are unrealistic in relation to modelling the human skin environment. The demonstration herein that the *camp2* gene appears to be evolving under functional constraints similar to housekeeping genes and is present and expressed in all strains, including those from genetic divisions containing health-associated lineages, suggests it may also be important for a commensal/normal existence as well as pathogenic lifestyle. The observation that the *camp2* gene may have been purged from other propionibacteria where, we can presume, it was not essential for adaptation, further supports a possible role in *P. acnes* survival on the human host. Interestingly, the double life of some bacterial housekeeping genes as virulence factors has been described [73]. In our opinion, therefore, proposed CAMP factor-related vaccines should be pursued with caution at this stage; indeed it is essential to demonstrate that immunisation with any target antigen does not disrupt commensal lineages within the skin microbiome that are associated with maintaining health via modulation of the innate immune host response and prevention of colonisation by more pathogenic microbes. As we describe in this paper, the investigation of *P. acnes* clonal diversification, combined with selection analysis and other *in silico*-based investigations, will enable informed choices to be made in relation to the further exploration of candidate vaccines.

## Materials and Methods

### Bacterial strains

All bacterial strains were maintained at −80°C in Brain Heart Infusion broth (Oxoid), containing 12% (v/v) glycerol, prior to analysis. *P. acnes* was cultured on Columbia agar base (Oxoid) supplemented with 5% (v/v) bovine blood, hemin (1 µg ml^−1^) and vitamin K1 (5 µg ml^−1^) for 72 h at 37°C in a vinyl anaerobic chamber (Coy Laboratories), under an atmosphere of 10% H_2_, 10% CO_2_ and 80% N_2_. A total of 87 isolates from acneic lesions (n = 24), healthy skin covering ‘acne-prone’ areas (n = 19), blood (n = 15), soft tissue infections (n = 27) and medical devices (n = 2) were analysed as part of this study (Table 2) [74].

### MLST analysis

Partial sequences of the housekeeping gene loci *atpD* (453 bp), *gmk* (400 bp), *guaA* (493 bp), *lepA* (452 bp), *sodA* (450 bp), *tly* (777 bp) and *camp2* (804 bp) were determined after PCR using primer pairs and amplification conditions previously described [9]. For *aroE*, a new set of forward (5′-ACCGATTAAGAGTGACTACC-3′) and reverse (5′-ACTCCTCGGAAATCTCTACA-3′) MLST primers were designed that target flanking sequences of the *aroE* open reading frame, and give rise to a 1102 bp amplicon (424bp after sequencing). PCR conditions were essentially as before, except that an annealing temperature of 56°C and MgCl_2_ concentration of 2.5 mM were used. PCR products were purified on MiniElute UF plates (Qiagen) and sequenced as detailed previously [9]. Novel alleles for each locus were assigned a new allele number, and distinct allelic profiles a new ST number, using the *P. acnes* MLST website (http://pubmlst.org/pacnes/). All MLST gene sequences are freely available for download at this site.

### Data analysis

The number of polymorphic sites, the average synonymous/non-synonymous site ration (*d_N_d_S_*), the nucleotide diversity per site (π), the average number of nucleotide differences per site (θ), Tajima *D* test and sliding window analysis were determined using DnaSp version 5 [75]. Mean pairwise distance (p-distance) and phylogenetic analysis using the Minimum Evolution algorithm were carried using MEGA v5.0. Split decomposition trees were generated using SplitsTree version 4.1 [76]. Evidence for recombination breakpoints was assessed using the genetic algorithm detection (GARD) method [77] prior to analysis of individual codons for positive or negative selection using FEL (Fixed Effects Likelihood) and REL (Random Effects Likelihood) methods with REV and HKY85 models of nucleotide substitution [41]. Index of association values (*I_A_*) were determined following the method of Haubold & Hudson [78] with LIAN v3.5 software. Clonal groups were identified using the eBURST v3 clustering algorithm which will also identify the most likely (i.e., parsimonious) ancestral ST within each clonal complex (http://www.mlst.net) [79]. Multiple amino acid alignments were created using PRofile ALIgNEment (PRALINE) (http://www.ibi.vu.nl/programs/pralinewww/) [80]. The presence of signal peptide cleavage sites in amino acid sequences was determined using SignalP v4.1 [81]. Simpson's discriminatory index (*D*), 95% confidence intervals (CI) and RAND values were determined as previously described [82].

### Multiple genome alignment

In order to investigate the rearrangement patterns and synteny between P. acnes (6609, GenBank accession no. CP002815) [83], *P. avidum* (ATCC25577, GenBank accession no. NZ_JH165060) and *P. humerusii* (P08, GenBank accession no. NZ_AFAM00000000) [55] genomes, we performed multiple alignments using the progressiveMauve algorithm (v2.3.1) [69]. We utilized the genome content distance matrix output of Mauve to quantify the differences in the aligned sequences.

### Estimation of recombination verses mutation rates

The per-allele and per-site recombination/mutation (*r/m*) ratio was estimated as previously described [84]. In brief, mutation events were presumed if the variant allele differed from the descendent SLV at one site, and this was not present in any other STs within the collection. Recombination events were identified if the variant allele differed at multiple sites or a single site which was also present in other STs of the collection, and not the result of vertical descent as clearly evident from inspection of an eBURST population diagram.

### Statistics

Data distributions were compared using the Chi Squared test, and statistical evidence of recombination using the phi test. P values for the discriminatory significance of the MLST schemes were calculated using the jack-knife pseudo-values re-sampling method.

## Supporting Information

Figure S1 **Alignment of** ***aroE*** **gene sequences representing all** ***P. acnes*** **phylogroups.** Sequences corresponding to the binding regions of the forward and reverse *aroE* amplification primers previously described for MLST analysis [9] are shaded. These primers were originally designed using whole genome data from the type IB strain KPA171202 [25]. Mismatches within the primer sequences are highlighted in red.
(PPT)

Figure S2 **Minimum evolution phylogenetic trees of (A)** ***recA*** **and (B)** ***rpoB*** **housekeeping genes from HMP taxa incorrectly classified as novel** ***Propionibacterium*** **species or** ***P.*** ***acnes***. Sequence input order was randomized, and bootstrapping resampling statistics were performed using 500 data sets. Bootstrap values (>50%) are shown on the arms of the tree. Homologous sequences from *P. humerusii* (P08; HL037PA1; HL044PA1) and *P. avidum* (ATCC25577) were used as outgroups. Horizontal bar represents genetic distance. HMP isolates CC003-HC2, 409-HCl, 434-HC2 and 5U42AFAA (highlighted in red), identified as potentially novel taxa, clearly cluster with *P. acnes*. HMP isolate SK182B-JCVI (highlighted in blue), classified as *P. acnes*, forms a distinct lineage from *P. acnes* strains; this was most pronounced with *rpoB*. Strains of *P. acnes* are represented by 266 (type IA_1_), P. acn33 (type IA_2_), KPA171202 (type IB), HL097PA1 (type IC), ATCC11828 (type II) and Asn12 (type III).
(PPT)

Figure S3 **Split decomposition analysis of all individual genes from the** ***P. acnes*** **MLST scheme.** No evidence of statistically significant recombination was identified in either the housekeeping or putative virulence gene sequences; although for loci such as *aroE*, *guaA* and *sodA* evidence of limited recombination events were identified due to the presence of interconnected pathways. P values were determined using the phi test.
(PPT)

Figure S4 **Amino acid alignments of CAMP factor homologues from** ***P. acnes*** **,** ***P. humerusii*** **and** ***P. avidum.*** Multiple sequence alignments were created using PRofile ALIgNEment (PRALINE) (http://www.ibi.vu.nl/programs/pralinewww/). (A) CAMP1 alignment, *P. acnes* versus *P. humerusii* (91% identity); (B) CAMP3 alignment, *P. acnes* versus *P. humerusii* (98% identity); (C) CAMP5 alignment, *P. acnes* versus *P. humerusii* (84% identity); (D) CAMP3 alignment, *P. acnes* versus *P. avidum* (92–93%); (E) CAMP5 alignment, *P. acnes* versus *P. avidum* (80% identity); (F) CAMP3 alignment, *P. humerusii* versus *P. avidum* (92–93% identity); (G) CAMP5 alignment, *P. humerusii* versus *P. avidum* (80% identity). *P. acnes* is represented by the type strain NCTC737 (type IA_1_; ST1; CC1); *P. humerusii* by P08, HL044PA1, HL037PA2 and HL037PA3; *P. avidum* by ATCC25577, 440671.
(TIF)

Figure S5 **Minimum evolution phylogenetic tree of 16S rDNA sequences for type strains of the genus** ***Propionibacterium*** **.** Sequence input order was randomized and bootstrapping resampling statistics were performed using 500 data sets. The 16S rDNA sequence from *Nocardia asteroides* was used as a distant outgroup to root the tree as it also belongs to the order *Actinomycetales*. Bootstrap values (>50%) are shown at each node of the tree. The blocks of species which could be analysed for the presence of CAMP factor homologues are highlighted. Horizontal bar represents genetic distance. Note, in *P humerusii*, the *camp4* gene generates a truncated product compared to the equivalent homologue in *P. acnes*.
(PPT)

Figure S6 **CAMP factor homologues appear located in similar regions within** ***P. acnes*** **,** ***P. avidum*** **and** ***P. humerusii*** **genomes.** Local collinear blocks are shown for *P. acnes* (6609, type IB, ST5, CC5; top), *P. avidum* (ATCC25577; middle) and *P. humerusii* (P08; bottom). Horizontally organised open rectangles show annotation of *P. acnes* genome; vertical open rectangles show the location of the given CAMP factor: (A) *camp1* (B) *camp2* (C) *camp3* (D) *camp4* (E) *camp5*.
(TIF)

Table S1 **Evaluation of MLST_4_ with MLST_8_ for the identification of phylogroup, CC and ST status of isolates.** By cross-referencing the four gene allelic profile to the full allelic profiles currently available within the growing *P. acnes* MLST database (http://pubmlst.org/pacnes/), it was possible to accurately determine the phylogroup (IA_1_, IA_2_, IB, IC, II and III) and CC/singleton identity. STs that could not be completely differentiated by MLST_4_ (16/91) are colour coded. Even in these cases, however, the MLST_4_ allelic profiles still correctly determined phylogroup and CC membership (S = singleton; CC = clonal complex).
(DOC)

Table S2 **MLST results for all 372** ***P. acnes*** **isolates analysed to date.** A total of 114 STs were generated based on the analysis of eight gene loci.
(DOC)
