# Supplementary material for: The Opportunistic Pathogen Propionibacterium acnes: Insights into Typing, Human Disease, Clonal Diversification and CAMP Factor Evolution
Source: PLoS One. 2013 Sep 13;8(9):e70897. doi: 10.1371/journal.pone.0070897 (PMC3772855; doi:10.1371/journal.pone.0070897)
Supplement: Figure S1 — Alignment of aroE gene sequences representing all P. acnes phylogroups. Sequences corresponding to the binding regions of the forward and reverse aroE amplification primers previously described for MLST analysis [9] are shaded. These primers were originally designed using whole genome data from the type IB strain KPA171202 [25]. Mismatches within the primer sequences are highlighted in red. (PPT) [file pone.0070897.s001.ppt]

## Slide 1
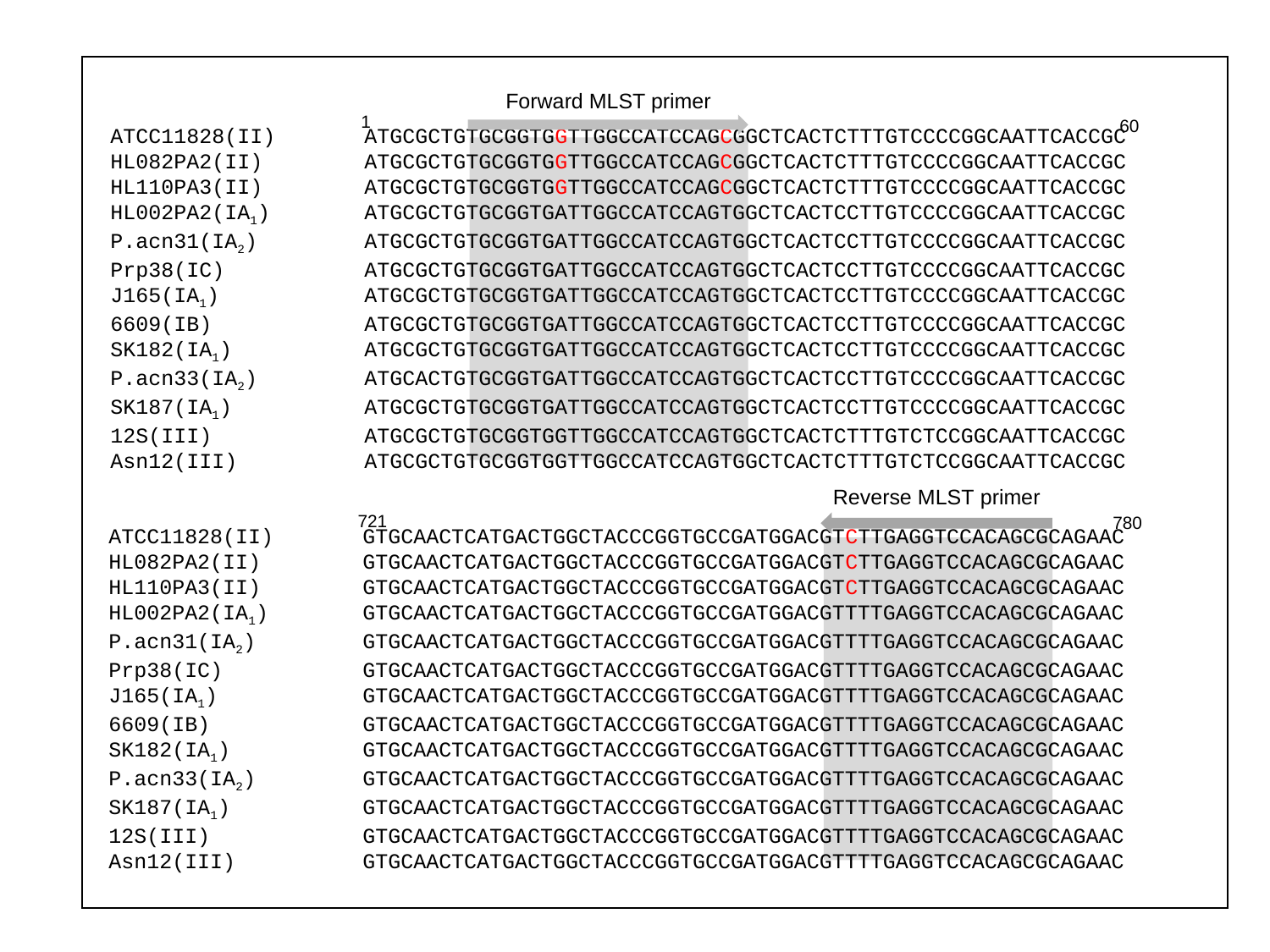

Forward MLST primer
1
60
ATCC11828(II)	ATGCGCTGTGCGGTGGTTGGCCATCCAGCGGCTCACTCTTTGTCCCCGGCAATTCACCGC
HL082PA2(II)	ATGCGCTGTGCGGTGGTTGGCCATCCAGCGGCTCACTCTTTGTCCCCGGCAATTCACCGC
HL110PA3(II)	ATGCGCTGTGCGGTGGTTGGCCATCCAGCGGCTCACTCTTTGTCCCCGGCAATTCACCGC
HL002PA2(IA1)	ATGCGCTGTGCGGTGATTGGCCATCCAGTGGCTCACTCCTTGTCCCCGGCAATTCACCGC
P.acn31(IA2)	ATGCGCTGTGCGGTGATTGGCCATCCAGTGGCTCACTCCTTGTCCCCGGCAATTCACCGC
Prp38(IC)		ATGCGCTGTGCGGTGATTGGCCATCCAGTGGCTCACTCCTTGTCCCCGGCAATTCACCGC
J165(IA1)		ATGCGCTGTGCGGTGATTGGCCATCCAGTGGCTCACTCCTTGTCCCCGGCAATTCACCGC
6609(IB)		ATGCGCTGTGCGGTGATTGGCCATCCAGTGGCTCACTCCTTGTCCCCGGCAATTCACCGC
SK182(IA1)		ATGCGCTGTGCGGTGATTGGCCATCCAGTGGCTCACTCCTTGTCCCCGGCAATTCACCGC
P.acn33(IA2)	ATGCACTGTGCGGTGATTGGCCATCCAGTGGCTCACTCCTTGTCCCCGGCAATTCACCGC
SK187(IA1)		ATGCGCTGTGCGGTGATTGGCCATCCAGTGGCTCACTCCTTGTCCCCGGCAATTCACCGC
12S(III)		ATGCGCTGTGCGGTGGTTGGCCATCCAGTGGCTCACTCTTTGTCTCCGGCAATTCACCGC
Asn12(III)	ATGCGCTGTGCGGTGGTTGGCCATCCAGTGGCTCACTCTTTGTCTCCGGCAATTCACCGC
Reverse MLST primer
721
780
ATCC11828(II) 	GTGCAACTCATGACTGGCTACCCGGTGCCGATGGACGTCTTGAGGTCCACAGCGCAGAAC
HL082PA2(II)	GTGCAACTCATGACTGGCTACCCGGTGCCGATGGACGTCTTGAGGTCCACAGCGCAGAAC
HL110PA3(II)	GTGCAACTCATGACTGGCTACCCGGTGCCGATGGACGTCTTGAGGTCCACAGCGCAGAAC
HL002PA2(IA1)	GTGCAACTCATGACTGGCTACCCGGTGCCGATGGACGTTTTGAGGTCCACAGCGCAGAAC
P.acn31(IA2)	GTGCAACTCATGACTGGCTACCCGGTGCCGATGGACGTTTTGAGGTCCACAGCGCAGAAC
Prp38(IC)		GTGCAACTCATGACTGGCTACCCGGTGCCGATGGACGTTTTGAGGTCCACAGCGCAGAAC
J165(IA1)		GTGCAACTCATGACTGGCTACCCGGTGCCGATGGACGTTTTGAGGTCCACAGCGCAGAAC
6609(IB)		GTGCAACTCATGACTGGCTACCCGGTGCCGATGGACGTTTTGAGGTCCACAGCGCAGAAC
SK182(IA1)		GTGCAACTCATGACTGGCTACCCGGTGCCGATGGACGTTTTGAGGTCCACAGCGCAGAAC
P.acn33(IA2)	GTGCAACTCATGACTGGCTACCCGGTGCCGATGGACGTTTTGAGGTCCACAGCGCAGAAC
SK187(IA1)		GTGCAACTCATGACTGGCTACCCGGTGCCGATGGACGTTTTGAGGTCCACAGCGCAGAAC
12S(III)		GTGCAACTCATGACTGGCTACCCGGTGCCGATGGACGTTTTGAGGTCCACAGCGCAGAAC
Asn12(III)	GTGCAACTCATGACTGGCTACCCGGTGCCGATGGACGTTTTGAGGTCCACAGCGCAGAAC
