# Supplementary material for: The Opportunistic Pathogen Propionibacterium acnes: Insights into Typing, Human Disease, Clonal Diversification and CAMP Factor Evolution
Source: PLoS One. 2013 Sep 13;8(9):e70897. doi: 10.1371/journal.pone.0070897 (PMC3772855; doi:10.1371/journal.pone.0070897)
Supplement: Figure S2 — Minimum evolution phylogenetic trees of (A) recA and (B) rpoB housekeeping genes from HMP taxa incorrectly classified as novel Propionibacterium species or P. acnes. Sequence input order was randomized, and bootstrapping resampling statistics were performed using 500 data sets. Bootstrap values (>50%) are shown on the arms of the tree. Homologous sequences from P. humerusii (P08; HL037PA1; HL044PA1) and P. avidum (ATCC25577) were used as outgroups. Horizontal bar represents genetic distance. HMP isolates CC003-HC2, 409-HCl, 434-HC2 and 5U42AFAA (highlighted in red), identified as potentially novel taxa, clearly cluster with P. acnes. HMP isolate SK182B-JCVI (highlighted in blue), classified as P. acnes, forms a distinct lineage from P. acnes strains; this was most pronounced with rpoB. Strains of P. acnes are represented by 266 (type IA1), P. acn33 (type IA2), KPA171202 (type IB), HL097PA1 (type IC), ATCC11828 (type II) and Asn12 (type III). (PPT) [file pone.0070897.s002.ppt]

## Slide 1
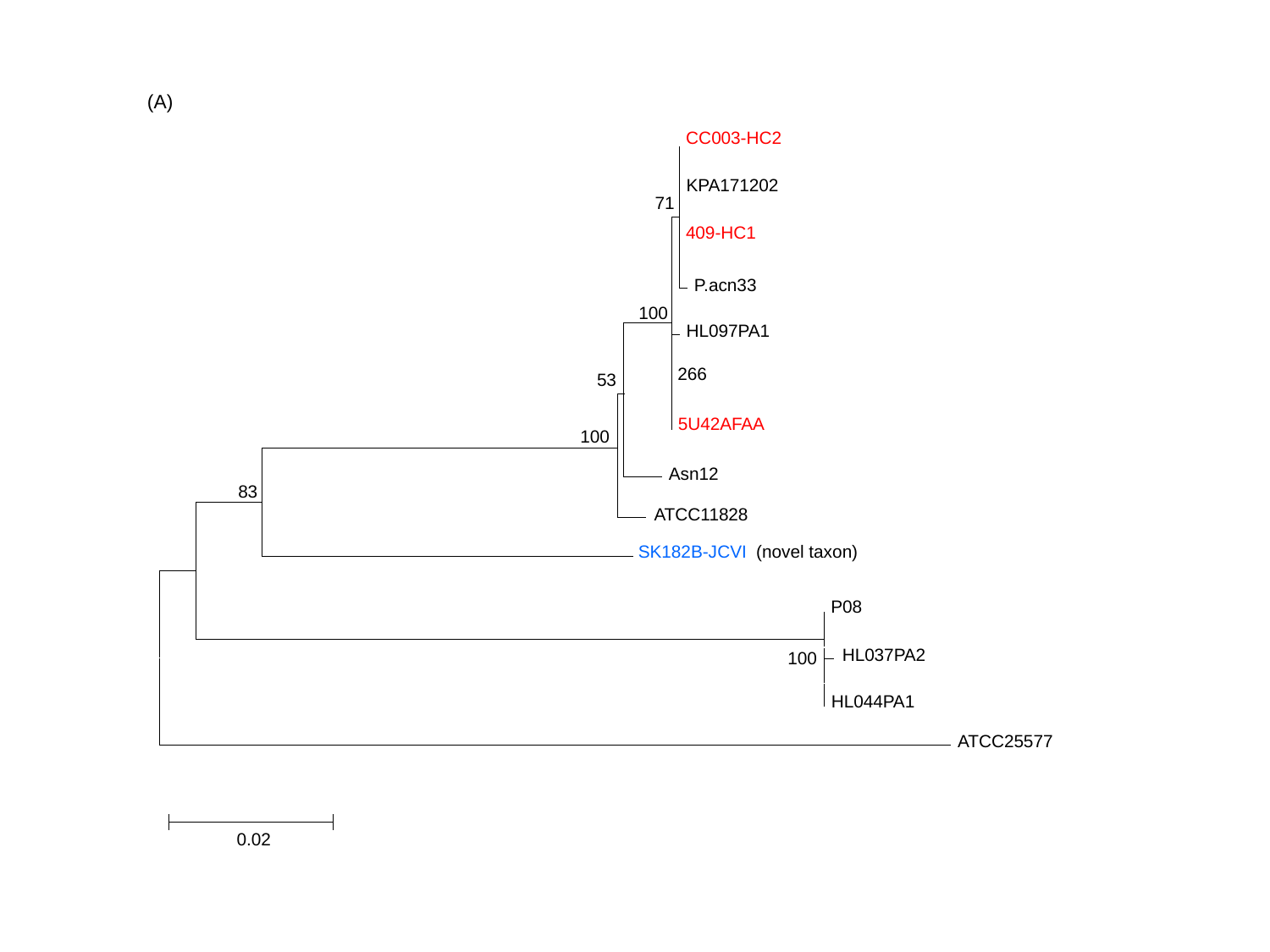

(A)
 CC003-HC2
 KPA171202
71
 409-HC1
 P.acn33
100
 HL097PA1
 266
53
 5U42AFAA
100
 Asn12
83
 ATCC11828
 SK182B-JCVI (novel taxon)
 P08
 HL037PA2
100
 HL044PA1
 ATCC25577
0.02

## Slide 2
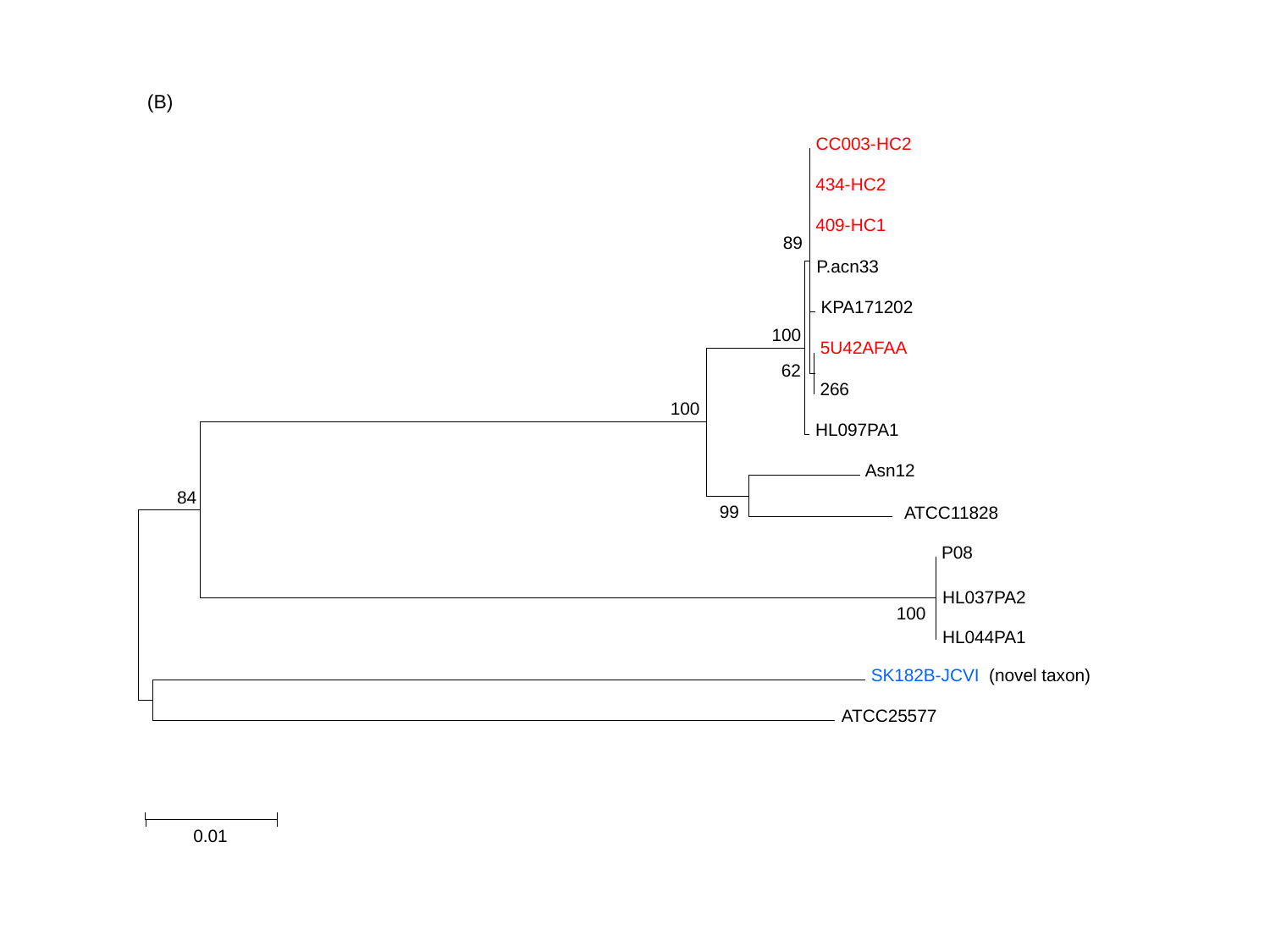

(B)
 CC003-HC2
 434-HC2
 409-HC1
89
 P.acn33
 KPA171202
100
 5U42AFAA
62
 266
100
 HL097PA1
 Asn12
84
99
 ATCC11828
 P08
 HL037PA2
100
 HL044PA1
 SK182B-JCVI (novel taxon)
 ATCC25577
0.01
