# Supplementary figures and images for: The Opportunistic Pathogen Propionibacterium acnes: Insights into Typing, Human Disease, Clonal Diversification and CAMP Factor Evolution
Source: PLoS One. 2013 Sep 13;8(9):e70897. doi: 10.1371/journal.pone.0070897 (PMC3772855; doi:10.1371/journal.pone.0070897)

## Slide 1
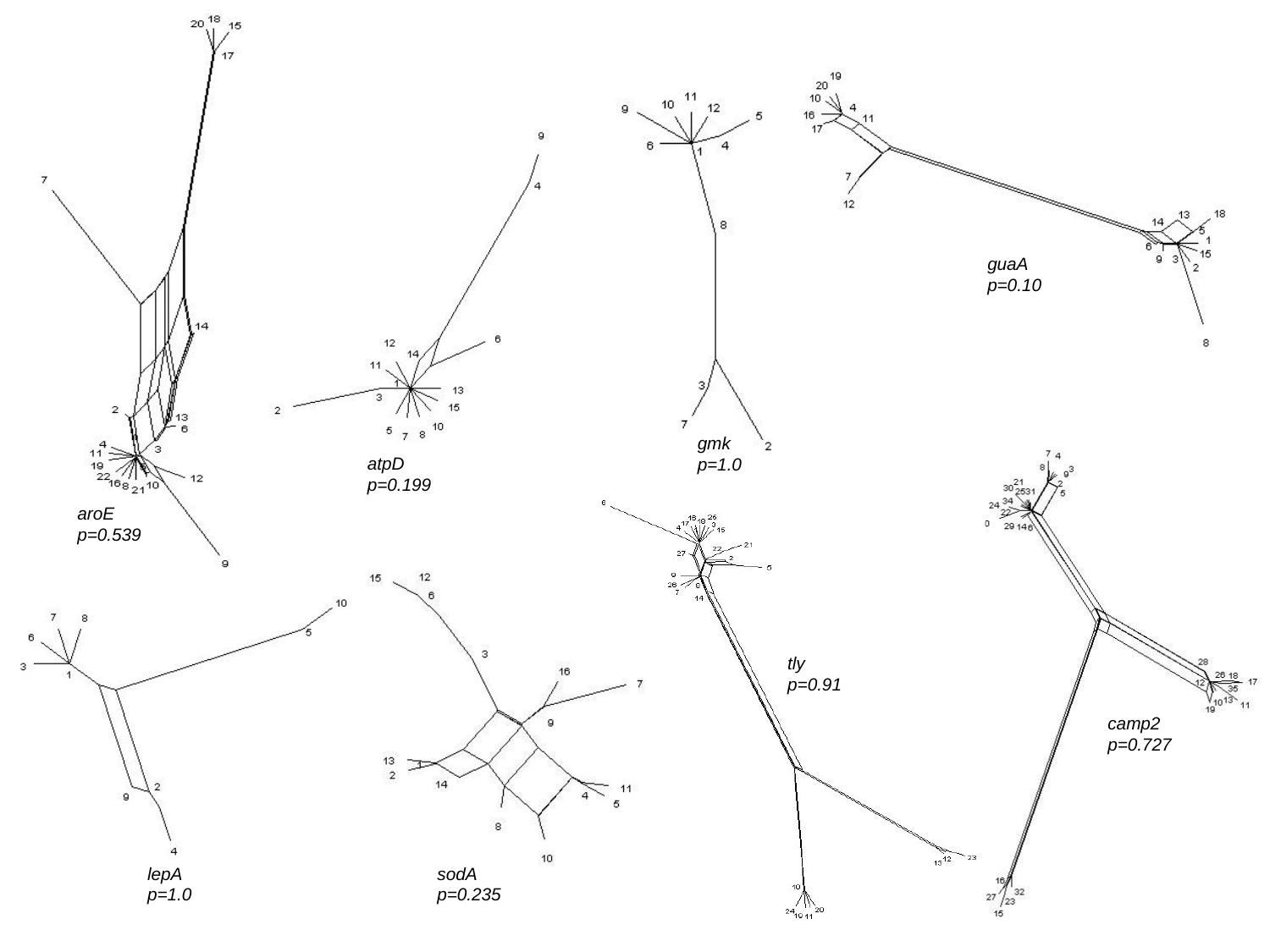

guaA
p=0.10
gmk
p=1.0
atpD
p=0.199
aroE
p=0.539
tly
p=0.91
camp2
p=0.727
lepA
p=1.0
sodA
p=0.235

Supplement: Figure S3 — Split decomposition analysis of all individual genes from the P. acnes MLST scheme. No evidence of statistically significant recombination was identified in either the housekeeping or putative virulence gene sequences; although for loci such as aroE, guaA and sodA evidence of limited recombination events were identified due to the presence of interconnected pathways. P values were determined using the phi test. (PPT) [file pone.0070897.s003.ppt]

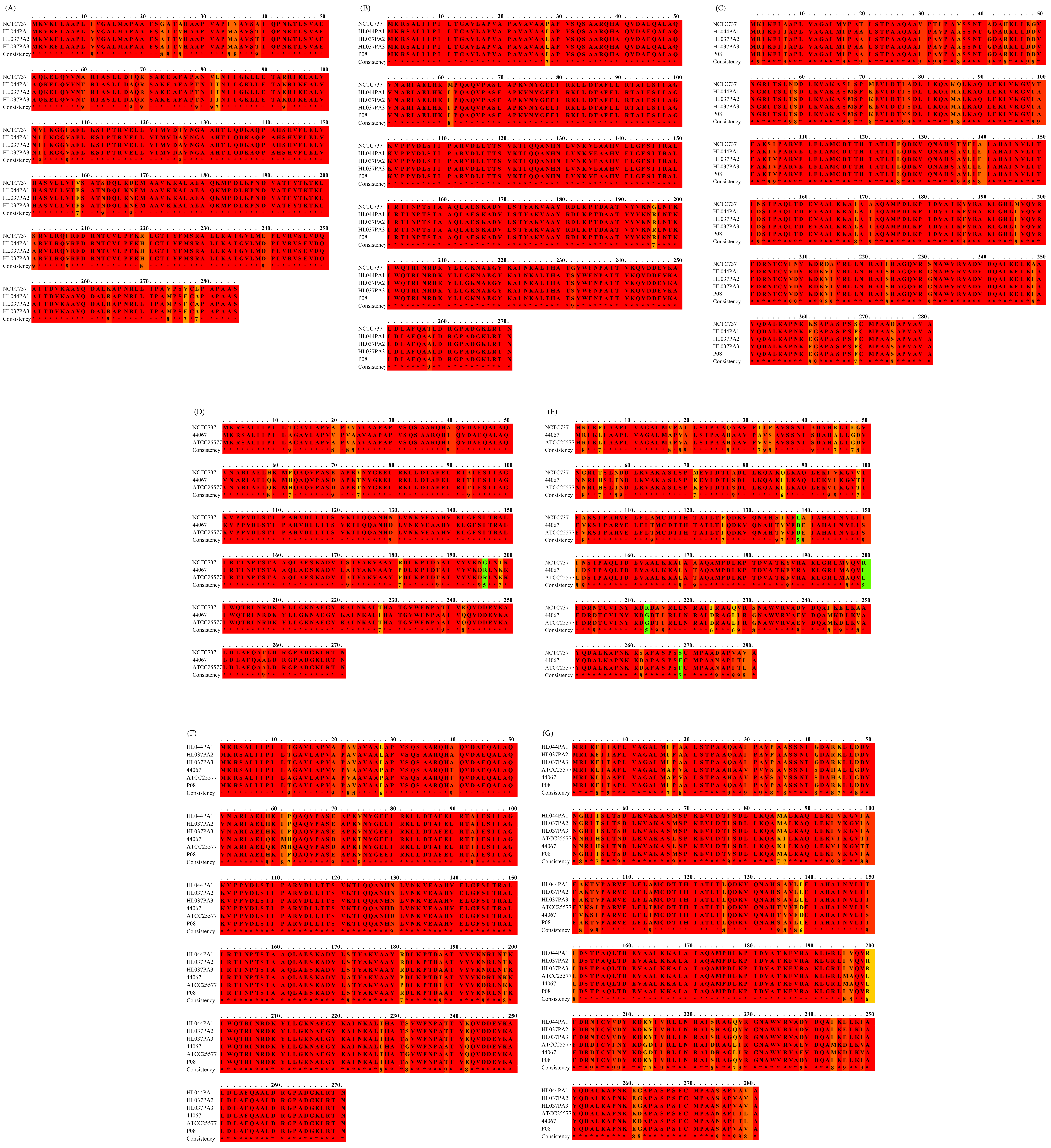

Supplement: Figure S4 — Amino acid alignments of CAMP factor homologues from P. acnes , P. humerusii and P. avidum. Multiple sequence alignments were created using PRofile ALIgNEment (PRALINE) (http://www.ibi.vu.nl/programs/pralinewww/). (A) CAMP1 alignment, P. acnes versus P. humerusii (91% identity); (B) CAMP3 alignment, P. acnes versus P. humerusii (98% identity); (C) CAMP5 alignment, P. acnes versus P. humerusii (84% identity); (D) CAMP3 alignment, P. acnes versus P. avidum (92–93%); (E) CAMP5 alignment, P. acnes versus P. avidum (80% identity); (F) CAMP3 alignment, P. humerusii versus P. avidum (92–93% identity); (G) CAMP5 alignment, P. humerusii versus P. avidum (80% identity). P. acnes is represented by the type strain NCTC737 (type IA1; ST1; CC1); P. humerusii by P08, HL044PA1, HL037PA2 and HL037PA3; P. avidum by ATCC25577, 440671. (TIF) [file pone.0070897.s004.tif]

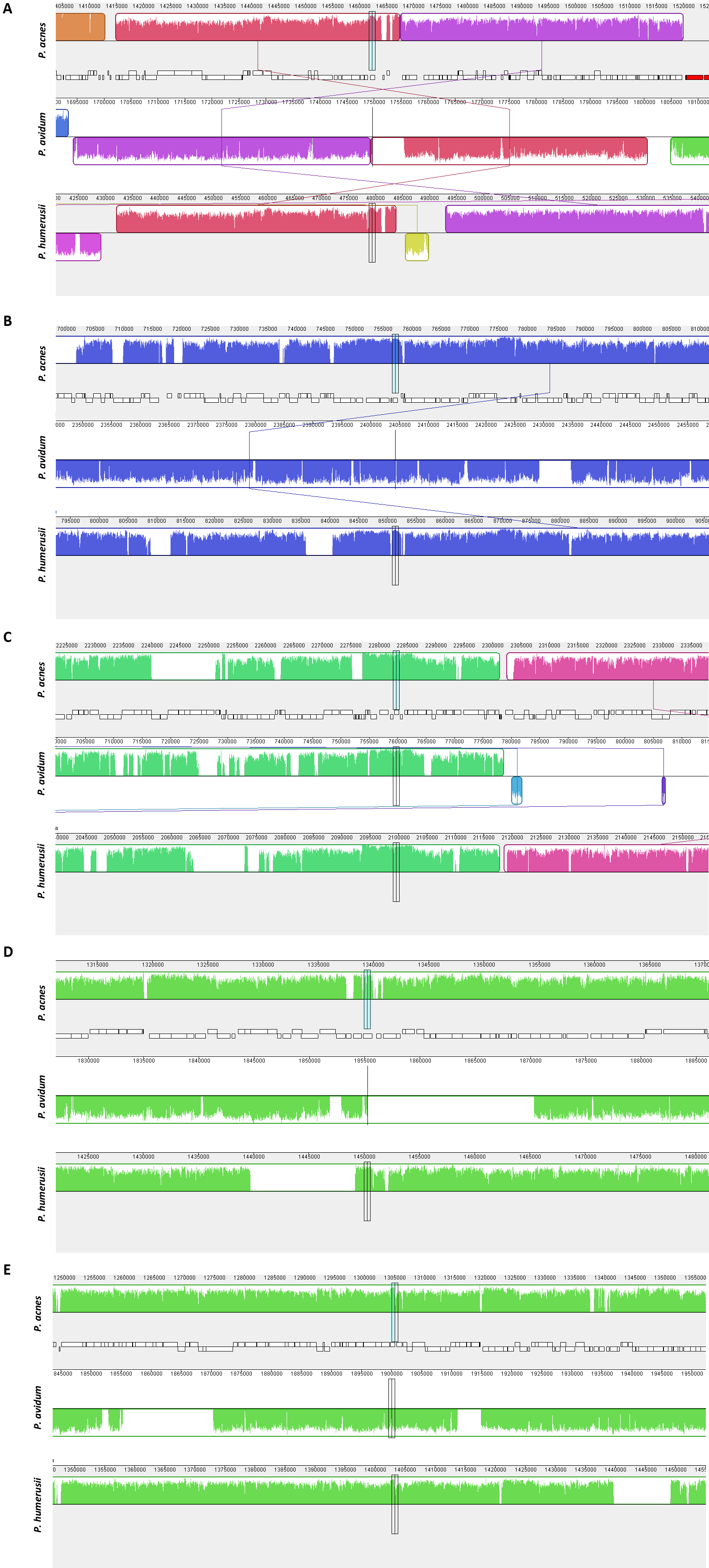

Supplement: Figure S6 — CAMP factor homologues appear located in similar regions within P. acnes , P. avidum and P. humerusii genomes. Local collinear blocks are shown for P. acnes (6609, type IB, ST5, CC5; top), P. avidum (ATCC25577; middle) and P. humerusii (P08; bottom). Horizontally organised open rectangles show annotation of P. acnes genome; vertical open rectangles show the location of the given CAMP factor: (A) camp1 (B) camp2 (C) camp3 (D) camp4 (E) camp5. (TIF) [file pone.0070897.s006.tif]
