# Supplementary material for: The Opportunistic Pathogen Propionibacterium acnes: Insights into Typing, Human Disease, Clonal Diversification and CAMP Factor Evolution
Source: PLoS One. 2013 Sep 13;8(9):e70897. doi: 10.1371/journal.pone.0070897 (PMC3772855; doi:10.1371/journal.pone.0070897)
Supplement: Figure S5 — Minimum evolution phylogenetic tree of 16S rDNA sequences for type strains of the genus Propionibacterium . Sequence input order was randomized and bootstrapping resampling statistics were performed using 500 data sets. The 16S rDNA sequence from Nocardia asteroides was used as a distant outgroup to root the tree as it also belongs to the order Actinomycetales. Bootstrap values (>50%) are shown at each node of the tree. The blocks of species which could be analysed for the presence of CAMP factor homologues are highlighted. Horizontal bar represents genetic distance. Note, in P humerusii, the camp4 gene generates a truncated product compared to the equivalent homologue in P. acnes. (PPT) [file pone.0070897.s005.ppt]

## Slide 1
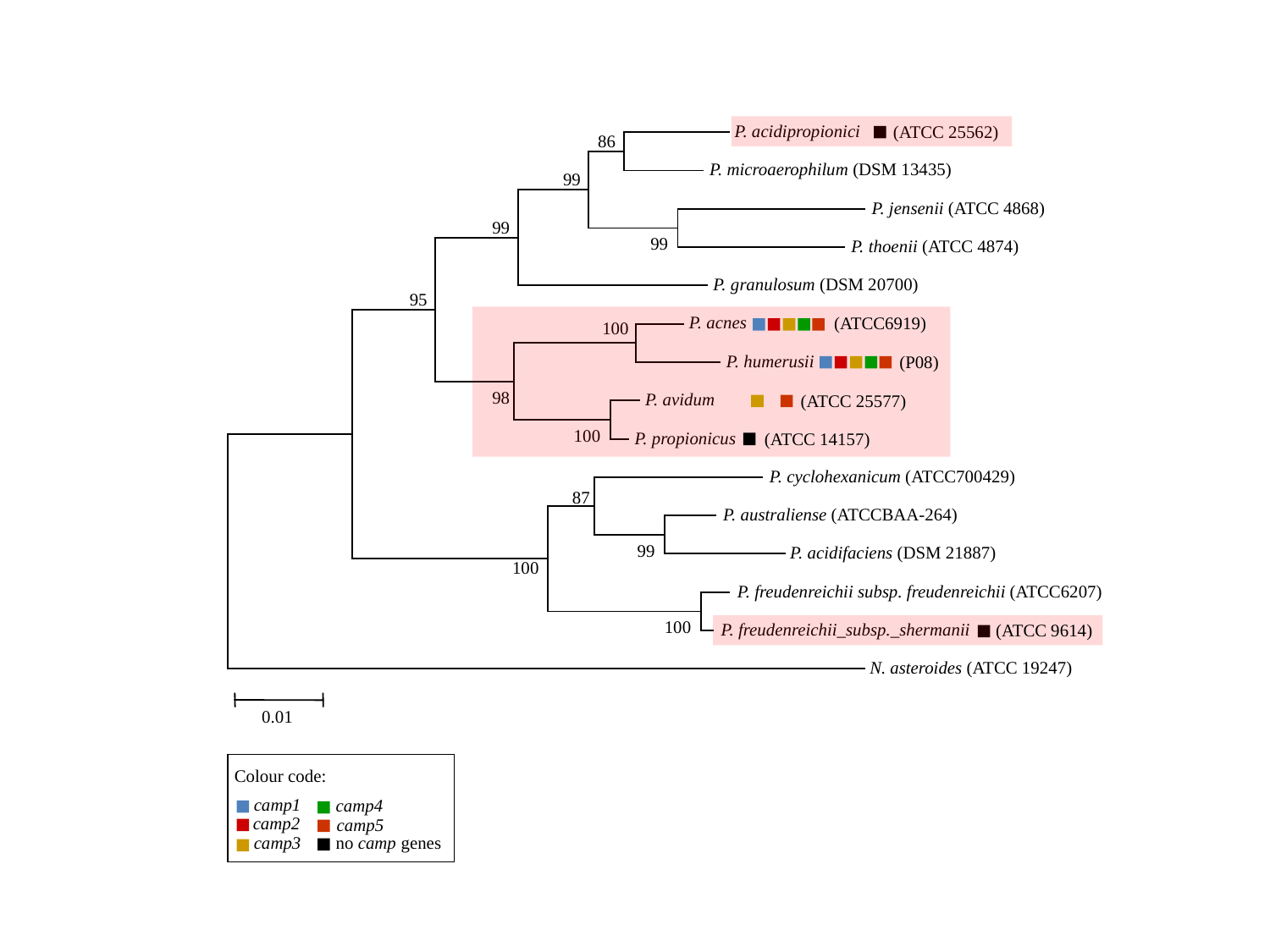

(ATCC 25562)
 P. acidipropionici
86
 P. microaerophilum (DSM 13435)
99
 P. jensenii (ATCC 4868)
99
99
 P. thoenii (ATCC 4874)
 P. granulosum (DSM 20700)
95
(ATCC6919)
 P. acnes
100
(P08)
 P. humerusii
(ATCC 25577)
98
 P. avidum
(ATCC 14157)
100
 P. propionicus
 P. cyclohexanicum (ATCC700429)
87
 P. australiense (ATCCBAA-264)
99
 P. acidifaciens (DSM 21887)
100
 P. freudenreichii subsp. freudenreichii (ATCC6207)
(ATCC 9614)
100
 P. freudenreichii_subsp._shermanii
 N. asteroides (ATCC 19247)
0.01
Colour code:
camp1
camp4
camp2
camp5
camp3
no camp genes
