# Supplementary material for: The Opportunistic Pathogen Propionibacterium acnes: Insights into Typing, Human Disease, Clonal Diversification and CAMP Factor Evolution
Source: PLoS One. 2013 Sep 13;8(9):e70897. doi: 10.1371/journal.pone.0070897 (PMC3772855; doi:10.1371/journal.pone.0070897)
Supplement: Table S1 — Evaluation of MLST4 with MLST8 for the identification of phylogroup, CC and ST status of isolates. By cross-referencing the four gene allelic profile to the full allelic profiles currently available within the growing P. acnes MLST database (http://pubmlst.org/pacnes/), it was possible to accurately determine the phylogroup (IA1, IA2, IB, IC, II and III) and CC/singleton identity. STs that could not be completely differentiated by MLST4 (16/91) are colour coded. Even in these cases, however, the MLST4 allelic profiles still correctly determined phylogroup and CC membership (S = singleton; CC = clonal complex). (DOC) [file pone.0070897.s007.doc]

| **MLST_8_**  **Allelic profile** | **MLST_8_**  **Phylotype/ CC/ ST** | **MLST_4_**  **Allelic profile** | **MLST_4_**  **Phylotype/ CC/ ST** |
| --- | --- | --- | --- |
| 1-1-1-3-1-1-1-1 | IA_1_/ CC1/ ST1 | 1-3-1-1 | IA_1_/ CC1/ ST1 |
| 1-1-1-5-1-4-8-2 | IA_2_/ CC2/ ST2 | 1-5-8-2 | IA_2_/ CC2/ ST2 |
| 1-1-1-3-1-1-2-2 | IA_1_/ CC3/ ST3 | 1-3-2-2 | IA_1_/ CC3/ ST3 |
| 1-1-1-3-1-1-8-6 | IA_1_/ CC4/ ST4 | 1-3-8-6 | IA_1_/ CC4/ ST4 |
| 1-1-1-4-1-4-8-6 | IB/ CC5/ ST5 | 1-4-8-6 | IB/ CC5/ ST5 |
| 17-4-2-4-2-3-10-10 | II/ CC6/ ST6 | 17-4-10-10 | II/ CC6/ ST6 |
| 15-4-2-4-2-3-10-10 | II/ CC6/ ST7 | 15-4-10-10 | II/ CC6/ ST7 |
| 1-1-1-3-1-1-3-1 | IA_1_/ CC1/ ST8 | 1-3-3-1 | IA_1_/ CC1/ ST8 |
| 1-1-1-3-1-1-4-1 | IA_1_/ CC1/ ST9 | 1-3-4-1 | IA_1_/ CC1/ ST9 |
| 1-1-1-3-1-1-2-4 | IA_1_/ CC3/ ST10 | 1-3-2-4 | IA_1_/ CC3/ ST10 |
| 1-1-1-3-1-1-5-2 | IA_1_/ CC3/ ST11 | 1-3-5-2 | IA_1_/ CC3/ ST11 |
| 1-1-1-4-1-4-8-31 | IB/ CC5/ ST12 | 1-4-8-31 | IB/ CC5/ ST12 |
| 1-1-1-3-1-1-7-6 | IA_1_/ CC4/ ST13 | 1-3-7-6 | IA_1_/ CC4/ ST13 |
| 1-1-1-3-1-14-2-3 | IA_1_/ CC3/ ST14 | 1-3-2-3 | IA_1_/ CC3/ ST14 |
| 1-1-10-3-1-1-6-1 | IA_1_/ CC1/ ST15 | 1-3-6-1 | IA_1_/ CC1/ ST15 |
| 1-1-10-3-1-1-1-1 | IA_1_/ CC1/ ST16 | 1-3-1-1 | IA_1_/ CC1/ ST1 |
| 1-10-1-3-1-1-2-2 | IA_1_/ CC3/ ST17 | 1-3-2-2 | IA_1_/ CC3/ ST3 |
| 8-1-1-3-1-1-2-5 | IA_1_/ CC3/ ST18 | 8-3-2-5 | IA_1_/ CC3/ ST18 |
| 16-1-1-15-1-4-8-6 | IA_1_/ S/ ST19 | 16-15-8-6 | IA_1_/ S/ ST19 |
| 5-1-1-3-1-1-1-1 | IA_1_/ CC1/ ST20 | 5-3-1-1 | IA_1_/ CC1/ ST20 |
| 1-1-1-1-1-1-8-6 | IA_1_/ CC4/ ST21 | 1-1-8-6 | IA_1_/ CC4 / ST21 |
| 1-1-1-5-3-5-8-7 | IA_2_/ S/ ST22 | 1-5-8-7 | IA_2_/ S/ ST22 |
| 1-8-1-5-3-5-8-8 | IA_2_/ S/ ST23 | 1-5-8-8 | IA_2_/ S/ ST23 |
| 1-1-1-5-1-4-8-9 | IA_2_/ CC2/ ST24 | 1-5-8-9 | IA_2_/ CC2/ ST24 |
| 17-9-2-4-2-3-10-10 | II/ CC6/ ST25 | 17-4-10-10 | II/ CC6/ ST6 |
| 17-4-2-17-2-3-11-11 | II/ S/ ST26 | 17-17-11-11 | II/ S/ ST26 |
| 17-4-2-4-9-12-10-13 | II/ S/ ST27 | 17-4-10-3 | II/ S/ ST27 |
| 17-4-2-16-2-12-10-12 | II/ CC72/ ST28 | 17-16-10-12 | II/ CC72/ ST28 |
| 1-13-1-3-1-1-2-5 | IA_1_/ S/ ST29 | 1-3-2-5 | IA_1_/ S/ ST29 |
| 17-4-2-4-2-6-10-12 | II/ CC72/ ST30 | 17-4-10-12 | II/ CC72/ ST30 |
| 1-1-1-3-1-1-2-3 | IA_1_/ CC3/ ST31 | 1-3-2-3 | IA_1_/ CC3/ ST14 |
| 7-6-3-7-5-9-12-15 | III/ CC77/ ST32 | 7-7-12-15 | III/ CC77/ ST32 |
| 7-6-3-7-5-9-13-16 | III/ CC77/ ST33 | 7-7-13-16 | III/ CC77/ ST33 |
| 1-1-1-1-1-1-8-22 | IA_1_/ CC4/ ST34 | 1-1-8-22 | IA_1_/ CC4/ ST34 |
| 1-2-1-2-1-1-2-2 | IA_1_/ S/ ST35 | 1-2-2-2 | IA_1_/ S/ ST35 |
| 1-1-1-13-1-4-8-2 | IA_2_/ CC2/ ST36 | 1-13-8-2 | IA_2_/ CC2/ ST36 |
| 1-1-1-3-1-2-15-1 | IA_1_/ S/ ST37 | 1-3-15-1 | IA_1_/ S/ ST37 |
| 1-1-1-3-1-1-16-1 | IA_1_/ CC1/ ST38 | 1-3-16-1 | IA_1_/ CC1/ ST38 |
| 1-1-1-3-1-1-2-20 | IA_1_/ CC3/ ST39 | 1-3-2-20 | IA_1_/ CC3/ ST39 |
| 1-5-1-3-1-1-2-2 | IA_1_/ CC3/ ST40 | 1-3-2-2 | IA_1_/ CC3/ ST3 |
| 1-1-1-6-1-1-1-1 | IA_1_/ CC1/ ST41 | 1-6-1-1 | IA_1_/ CC1/ ST41 |
| 1-1-1-4-1-4-8-21 | IB/ CC5/ ST42 | 1-4-8-21 | IB/ CC5/ ST42 |
| 8-1-1-3-1-1-2-2 | IA_1_/ CC3/ ST43 | 8-3-2-2 | IA_1_/ CC3/ ST43 |
| 11-1-1-3-1-1-1-1 | IA_1_/ CC1/ ST44 | 11-3-1-1 | IA_1_/ CC1/ ST44 |
| 1-1-6-3-1-1-1-1 | IA_1_/ CC1/ ST45 | 1-3-1-1 | IA_1_/ CC1/ ST1 |
| 1-1-1-14-1-1-3-24 | IA_1_/ S/ ST46 | 1-14-3-24 | IA_1_/ S/ ST46 |
| 4-1-1-3-1-1-1-1 | IA_1_/ CC1/ ST47 | 4-3-1-1 | IA_1_/ CC1/ ST47 |
| 1-3-1-2-1-1-1-1 | IA_1_/ S/ ST48 | 1-2-1-1 | IA_1_/ S/ ST48 |
| 1-1-1-9-1-1-1-1 | IA_1_/ CC1/ ST49 | 1-9-1-1 | IA_1_/ CC1/ ST49 |
| 12-1-1-3-1-1-1-1 | IA_1_/ CC1/ ST50 | 12-3-1-1 | IA_1_/ CC1/ ST50 |
| 1-1-9-4-1-11-8-25 | IB/ S/ ST51 | 1-4-8-25 | IB/ S/ ST51 |
| 1-1-1-3-8-1-8-6 | IA_1_/ CC4/ ST52 | 1-3-8-6 | IA_1_/ CC4/ ST4 |
| 1-1-9-4-1-4-8-6 | IB/ CC5/ ST53 | 1-4-8-6 | IB/ CC5/ ST5 |
| 1-1-1-3-1-13-18-1 | IA_1_/ S/ ST54 | 1-3-18-1 | IA_1_/ S// ST54 |
| 1-12-1-3-1-1-8-6 | IA_1_/ CC4/ ST55 | 1-3-8-6 | IA_1_/ CC4/ ST4 |
| 1-1-12-4-1-4-8-6 | IB/ CC5/ ST56 | 1-4-8-6 | IB/ CC5/ ST5 |
| 1-1-1-18-1-4-8-2 | IA_2_/ CC2/ ST57 | 1-18-8-2 | IA_2_/ CC2/ ST57 |
| 2-4-2-4-2-3-10-26 | II/ S/ ST58 | 2-4-10-26 | II/ S/ ST58 |
| 13-4-2-4-2-3-10-10 | II/ CC6/ ST59 | 13-4-10-10 | II/ CC6/ ST59 |
| 3-4-2-4-2-3-10-10 | II/ CC6/ ST60 | 3-4-10-10 | II/ CC6/ ST60 |
| 1-4-2-4-2-3-10-10 | II/ CC6/ ST61 | 1-4-10-10 | II/ CC6/ ST61 |
| 3-4-2-10-4-6-10-18 | II/ S/ ST62 | 3-10-10-18 | II/ S/ ST62 |
| 14-4-2-4-4-5-10-18 | II/ CC72/ ST63 | 14-4-10-18 | II/ CC72/ ST63 |
| 6-4-2-4-4-6-19-12 | II/ S/ ST64 | 6-4-19-12 | II/ S/ ST64 |
| 17-4-2-4-2-3-10-19 | II/ CC6/ ST65 | 17-4-10-19 | II/ CC6/ ST65 |
| 17-4-2-4-2-3-10-11 | II/ CC6/ ST66 | 17-4-10-11 | II/ CC6/ ST66 |
| 17-4-2-4-4-6-10-17 | II/ CC72/ ST67 | 17-4-10-17 | II/ CC72/ ST67 |
| 17-4-2-4-4-6-10-18 | II/ CC72/ ST68 | 17-4-10-18 | II/ CC72/ ST68 |
| 17-4-2-4-4-6-10-12 | II/ CC72/ ST69 | 17-4-10-12 | II/ CC72/ ST30 |
| 9-1-4-8-6-8-14-14 | IC/ CC107/ ST70 | 9-8-14-14 | IC/ CC107/ ST70 |
| 18-4-2-4-2-6-20-12 | II/ S/ ST71 | 18-4-20-12 | II/ S/ ST71 |
| 17-4-2-4-2-12-10-12 | II/ CC72/ ST72 | 17-4-10-12 | II/ CC72/ ST30 |
| 7-6-3-11-5-9-13-16 | III/ CC77/ ST73 | 7-11-13-16 | III/ CC77/ ST73 |
| 7-6-3-7-5-9-13-27 | III/ CC77/ ST74 | 7-7-13-27 | III/ CC77/ ST74 |
| 7-6-3-7-5-9-12-23 | III/ CC77/ ST75 | 7-7-12-23 | III/ CC77/ ST75 |
| 7-6-3-7-10-9-12-16 | III/ CC77/ ST76 | 7-7-12-16 | III/ CC77/ ST76 |
| 7-6-3-7-5-9-12-16 | III/ CC77/ ST77 | 7-7-12-16 | III/ CC77/ ST76 |
| 1-1-1-4-1-4-8-29 | IB/ CC5/ ST78 | 1-4-8-29 | IB/ CC5/ ST78 |
| 17-4-2-4-2-12-10-28 | IB/ CC5/ ST79 | 17-4-10-28 | IB/ CC5/ ST79 |
| 1-11-1-3-1-1-8-6 | IA_1_/ CC4/ ST80 | 1-3-8-6 | IA_1_/ CC4/ ST4 |
| 7-6-7-7-5-9-12-16 | III/ CC77/ ST81 | 7-7-12-16 | III/ CC77/ ST76 |
| 1-1-1-3-1-1-21-2 | IA_1_/ CC3/ ST82 | 1-3-21-2 | IA_1_/ CC3/ ST82 |
| 1-1-1-3-1-1-8-30 | IA_1_/ CC4/ ST83 | 1-3-8-30 | IA_1_/ CC4/ ST83 |
| 19-1-1-4-1-4-8-21 | IB/ CC5/ ST84 | 19-4-8-21 | IB/ CC5/ ST84 |
| 9-1-5-8-6-8-14-6 | IC/ CC107/ ST85 | 9-8-14-6 | IC/ CC107/ ST85 |
| 1-1-1-3-7-1-22-2 | IA_1_/ S/ ST86 | 1-3-22-2 | IA_1_/ S/ ST86 |
| 1-1-11-3-1-1-1-1 | IA_1_/ CC1/ ST87 | 1-3-1-1 | IA_1_/ CC1/ ST1 |
| 1-1-1-3-1-1-17-33 | IA_1_/ S/ ST88 | 1-3-17-33 | IA_1_/ S ST88 |
| 1-1-1-4-1-4-8-1 | IB/ CC5/ ST89 | 1-4-8-1 | IB/ CC5/ ST89 |
| 7-6-3-7-5-7-23-32 | III/ S/ ST90 | 7-7-23-32 | III/ S/ ST90 |
| 1-1-1-5-1-4-9-2 | IA_2_/ CC2/ ST91 | 1-5-9-2 | IA_2_/ CC2/ ST91 |
