# Supplementary material for: The Opportunistic Pathogen Propionibacterium acnes: Insights into Typing, Human Disease, Clonal Diversification and CAMP Factor Evolution
Source: PLoS One. 2013 Sep 13;8(9):e70897. doi: 10.1371/journal.pone.0070897 (PMC3772855; doi:10.1371/journal.pone.0070897)
Supplement: Table S2 — MLST results for all 372 P. acnes isolates analysed to date. A total of 114 STs were generated based on the analysis of eight gene loci. (DOC) [file pone.0070897.s008.doc]

|  |  |  | **Allelic profile** | | | | | | | |  |
| --- | --- | --- | --- | --- | --- | --- | --- | --- | --- | --- | --- |
| **Isolate** | **Source** | **Phylogroup** | ***aroE*** | ***atpD*** | ***gmk*** | ***guaA*** | ***lepA*** | ***sodA*** | ***tly*** | ***camp2*** | **ST** |
| NCTC737^a^ | Acne | IA_1_ | 1 | 1 | 1 | 3 | 1 | 1 | 1 | 1 | 1 |
| XnT2 | Fatal head granuloma | IA_1_ | 1 | 1 | 1 | 3 | 1 | 1 | 1 | 1 | 1 |
| XnH | Fatal head granuloma | IA_1_ | 1 | 1 | 1 | 3 | 1 | 1 | 1 | 1 | 1 |
| PRP-113 | Acne | IA_1_ | 1 | 1 | 1 | 3 | 1 | 1 | 1 | 1 | 1 |
| PV13 | Acne | IA_1_ | 1 | 1 | 1 | 3 | 1 | 1 | 1 | 1 | 1 |
| PV20 | Acne | IA_1_ | 1 | 1 | 1 | 3 | 1 | 1 | 1 | 1 | 1 |
| PV37 | Acne | IA_1_ | 1 | 1 | 1 | 3 | 1 | 1 | 1 | 1 | 1 |
| PV41 | Acne | IA_1_ | 1 | 1 | 1 | 3 | 1 | 1 | 1 | 1 | 1 |
| PV58 | Acne | IA_1_ | 1 | 1 | 1 | 3 | 1 | 1 | 1 | 1 | 1 |
| PV109 | Acne | IA_1_ | 1 | 1 | 1 | 3 | 1 | 1 | 1 | 1 | 1 |
| PV113 | Acne | IA_1_ | 1 | 1 | 1 | 3 | 1 | 1 | 1 | 1 | 1 |
| PV138 | Acne | IA_1_ | 1 | 1 | 1 | 3 | 1 | 1 | 1 | 1 | 1 |
| W513 | Dental | IA_1_ | 1 | 1 | 1 | 3 | 1 | 1 | 1 | 1 | 1 |
| P.acn11 | Aqueous humour | IA_1_ | 1 | 1 | 1 | 3 | 1 | 1 | 1 | 1 | 1 |
| P.acn12 | Corneal scrape | IA_1_ | 1 | 1 | 1 | 3 | 1 | 1 | 1 | 1 | 1 |
| P.acn14 | Corneal scrape | IA_1_ | 1 | 1 | 1 | 3 | 1 | 1 | 1 | 1 | 1 |
| P.acn15 | Corneal scrape | IA_1_ | 1 | 1 | 1 | 3 | 1 | 1 | 1 | 1 | 1 |
| P.acn34 | Corneal scrape | IA_1_ | 1 | 1 | 1 | 3 | 1 | 1 | 1 | 1 | 1 |
| P.acn26 | Eye | IA_1_ | 1 | 1 | 1 | 3 | 1 | 1 | 1 | 1 | 1 |
| P.acn23 | Vitreous humour | IA_1_ | 1 | 1 | 1 | 3 | 1 | 1 | 1 | 1 | 1 |
| W1973 | Dental | IA_1_ | 1 | 1 | 1 | 3 | 1 | 1 | 1 | 1 | 1 |
| J165 | Skin | IA_1_ | 1 | 1 | 1 | 3 | 1 | 1 | 1 | 1 | 1 |
| 889^b^ | Endocarditis | IA_1_ | 1 | 1 | 1 | 3 | 1 | 1 | 1 | 1 | 1 |
| GN-2^c^ | Acne | IA_1_ | 1 | 1 | 1 | 3 | 1 | 1 | 1 | 1 | 1 |
| GP-2^c^ | Acne | IA_1_ | 1 | 1 | 1 | 3 | 1 | 1 | 1 | 1 | 1 |
| P-13 | Acne | IA_1_ | 1 | 1 | 1 | 3 | 1 | 1 | 1 | 1 | 1 |
| 411.2 | Acne | IA_1_ | 1 | 1 | 1 | 3 | 1 | 1 | 1 | 1 | 1 |
| PRP-004 | Acne | IA_1_ | 1 | 1 | 1 | 3 | 1 | 1 | 1 | 1 | 1 |
| VA1/5 | Skin | IA_1_ | 1 | 1 | 1 | 3 | 1 | 1 | 1 | 1 | 1 |
| VA1/10 | Skin | IA_1_ | 1 | 1 | 1 | 3 | 1 | 1 | 1 | 1 | 1 |
| VA1/15 | Skin | IA_1_ | 1 | 1 | 1 | 3 | 1 | 1 | 1 | 1 | 1 |
| VA1/27 | Skin | IA_1_ | 1 | 1 | 1 | 3 | 1 | 1 | 1 | 1 | 1 |
| VA2/10 | Skin | IA_1_ | 1 | 1 | 1 | 3 | 1 | 1 | 1 | 1 | 1 |
| VA3/1 | Skin | IA_1_ | 1 | 1 | 1 | 3 | 1 | 1 | 1 | 1 | 1 |
| 317669 | Skin | IA_1_ | 1 | 1 | 1 | 3 | 1 | 1 | 1 | 1 | 1 |
| 33257 | Acne | IA_1_ | 1 | 1 | 1 | 3 | 1 | 1 | 1 | 1 | 1 |
| HL036PA3 | Skin | IA_1_ | 1 | 1 | 1 | 3 | 1 | 1 | 1 | 1 | 1 |
| HL072PA2 | Acne | IA_1_ | 1 | 1 | 1 | 3 | 1 | 1 | 1 | 1 | 1 |
| HL046PA2 | Acne | IA_1_ | 1 | 1 | 1 | 3 | 1 | 1 | 1 | 1 | 1 |
| HL087PA2 | Acne | IA_1_ | 1 | 1 | 1 | 3 | 1 | 1 | 1 | 1 | 1 |
| HL002PA3 | Acne | IA_1_ | 1 | 1 | 1 | 3 | 1 | 1 | 1 | 1 | 1 |
| HL027PA2 | Skin | IA_1_ | 1 | 1 | 1 | 3 | 1 | 1 | 1 | 1 | 1 |
| HL036PA2 | Skin | IA_1_ | 1 | 1 | 1 | 3 | 1 | 1 | 1 | 1 | 1 |
| HL036PA1 | Skin | IA_1_ | 1 | 1 | 1 | 3 | 1 | 1 | 1 | 1 | 1 |
| HL063PA1 | Skin | IA_1_ | 1 | 1 | 1 | 3 | 1 | 1 | 1 | 1 | 1 |
| HL013PA2 | Acne | IA_1_ | 1 | 1 | 1 | 3 | 1 | 1 | 1 | 1 | 1 |
| HL072PA1 | Acne | IA_1_ | 1 | 1 | 1 | 3 | 1 | 1 | 1 | 1 | 1 |
| HL002PA2 | Acne | IA_1_ | 1 | 1 | 1 | 3 | 1 | 1 | 1 | 1 | 1 |
| 15.2.L1 | [16] | IA_1_ | 1 | 1 | 1 | 3 | 1 | 1 | 1 | 1 | 1 |
| 27.1.R1 | [16] | IA_1_ | 1 | 1 | 1 | 3 | 1 | 1 | 1 | 1 | 1 |
| KF16 | Acne | IA_1_ | 1 | 1 | 1 | 3 | 1 | 1 | 1 | 1 | 1 |
| HDN-1 | Acne | IA_1_ | 1 | 1 | 1 | 3 | 1 | 1 | 1 | 1 | 1 |
| IT100EA | Acne | IA_1_ | 1 | 1 | 1 | 3 | 1 | 1 | 1 | 1 | 1 |
| SP64E | Acne | IA_1_ | 1 | 1 | 1 | 3 | 1 | 1 | 1 | 1 | 1 |
| 55835 | Cystitis | IA_1_ | 1 | 1 | 1 | 3 | 1 | 1 | 1 | 1 | 1 |
| 3632 | Blood | IA_1_ | 1 | 1 | 1 | 3 | 1 | 1 | 1 | 1 | 1 |
| 4579 | Skin | IA_1_ | 1 | 1 | 1 | 3 | 1 | 1 | 1 | 1 | 1 |
| 12604 | Pleural aspirate | IA_1_ | 1 | 1 | 1 | 3 | 1 | 1 | 1 | 1 | 1 |
| 16634 | Acne | IA_1_ | 1 | 1 | 1 | 3 | 1 | 1 | 1 | 1 | 1 |
| 63006 | Acne | IA_1_ | 1 | 1 | 1 | 3 | 1 | 1 | 1 | 1 | 1 |
| 19107 | Abscess | IA_1_ | 1 | 1 | 1 | 3 | 1 | 1 | 1 | 1 | 1 |
| 20550 | Intraabdominal | IA_1_ | 1 | 1 | 1 | 3 | 1 | 1 | 1 | 1 | 1 |
| M43 | Blood | IA_1_ | 1 | 1 | 1 | 3 | 1 | 1 | 1 | 1 | 1 |
| 50460 | Acne | IA_1_ | 1 | 1 | 1 | 3 | 1 | 1 | 1 | 1 | 1 |
| 64745 | Abscess | IA_1_ | 1 | 1 | 1 | 3 | 1 | 1 | 1 | 1 | 1 |
| 2-2-1 | Acne | IA_1_ | 1 | 1 | 1 | 3 | 1 | 1 | 1 | 1 | 1 |
| 2-4-6 | Skin | IA_1_ | 1 | 1 | 1 | 3 | 1 | 1 | 1 | 1 | 1 |
| P.acn10 | Corneal scrape | IA_2_ | 1 | 1 | 1 | 5 | 1 | 4 | 8 | 2 | 2 |
| P.acn24 | Aqueous humour | IA_2_ | 1 | 1 | 1 | 5 | 1 | 4 | 8 | 2 | 2 |
| P.acn33 | Aqueous humour | IA_2_ | 1 | 1 | 1 | 5 | 1 | 4 | 8 | 2 | 2 |
| P.acn35 | Corneal scrape | IA_2_ | 1 | 1 | 1 | 5 | 1 | 4 | 8 | 2 | 2 |
| P.acn39 | Aqueous humour | IA_2_ | 1 | 1 | 1 | 5 | 1 | 4 | 8 | 2 | 2 |
| 38A | Skin | IA_2_ | 1 | 1 | 1 | 5 | 1 | 4 | 8 | 2 | 2 |
| 1A | Skin | IA_2_ | 1 | 1 | 1 | 5 | 1 | 4 | 8 | 2 | 2 |
| HL059PA2 | Skin | IA_2_ | 1 | 1 | 1 | 5 | 1 | 4 | 8 | 2 | 2 |
| HL037PA1 | Skin | IA_2_ | 1 | 1 | 1 | 5 | 1 | 4 | 8 | 2 | 2 |
| HL027PA1 | Skin | IA_2_ | 1 | 1 | 1 | 5 | 1 | 4 | 8 | 2 | 2 |
| HL013PA1 | Acne | IA_2_ | 1 | 1 | 1 | 5 | 1 | 4 | 8 | 2 | 2 |
| HL083PA2 | Acne | IA_2_ | 1 | 1 | 1 | 5 | 1 | 4 | 8 | 2 | 2 |
| HL087PA1 | Acne | IA_2_ | 1 | 1 | 1 | 5 | 1 | 4 | 8 | 2 | 2 |
| HL059PA1 | Skin | IA_2_ | 1 | 1 | 1 | 5 | 1 | 4 | 8 | 2 | 2 |
| HL087PA3 | Acne | IA_2_ | 1 | 1 | 1 | 5 | 1 | 4 | 8 | 2 | 2 |
| HL046PA1 | Acne | IA_2_ | 1 | 1 | 1 | 5 | 1 | 4 | 8 | 2 | 2 |
| HL050PA3 | Skin | IA_2_ | 1 | 1 | 1 | 5 | 1 | 4 | 8 | 2 | 2 |
| HL025PA2 | Skin | IA_2_ | 1 | 1 | 1 | 5 | 1 | 4 | 8 | 2 | 2 |
| HL005PA4 | Skin | IA_2_ | 1 | 1 | 1 | 5 | 1 | 4 | 8 | 2 | 2 |
| HL002PA1 | Acne | IA_2_ | 1 | 1 | 1 | 5 | 1 | 4 | 8 | 2 | 2 |
| 1-1-2 | Acne | IA_2_ | 1 | 1 | 1 | 5 | 1 | 4 | 8 | 2 | 2 |
| 1-3-2 | Acne | IA_2_ | 1 | 1 | 1 | 5 | 1 | 4 | 8 | 2 | 2 |
| PV10 | Acne | IA_1_ | 1 | 1 | 1 | 3 | 1 | 1 | 2 | 2 | 3 |
| PV84 | Acne | IA_1_ | 1 | 1 | 1 | 3 | 1 | 1 | 2 | 2 | 3 |
| P.acn28 | Corneal scrape | IA_1_ | 1 | 1 | 1 | 3 | 1 | 1 | 2 | 2 | 3 |
| 308.2 | Acne | IA_1_ | 1 | 1 | 1 | 3 | 1 | 1 | 2 | 2 | 3 |
| 322.2 | Acne | IA_1_ | 1 | 1 | 1 | 3 | 1 | 1 | 2 | 2 | 3 |
| 401.5 | Acne | IA_1_ | 1 | 1 | 1 | 3 | 1 | 1 | 2 | 2 | 3 |
| 416.1 | Acne | IA_1_ | 1 | 1 | 1 | 3 | 1 | 1 | 2 | 2 | 3 |
| 423 | Acne | IA_1_ | 1 | 1 | 1 | 3 | 1 | 1 | 2 | 2 | 3 |
| 423.3 | Acne | IA_1_ | 1 | 1 | 1 | 3 | 1 | 1 | 2 | 2 | 3 |
| 425 | Acne | IA_1_ | 1 | 1 | 1 | 3 | 1 | 1 | 2 | 2 | 3 |
| 429 | Acne | IA_1_ | 1 | 1 | 1 | 3 | 1 | 1 | 2 | 2 | 3 |
| R11883 | Blood culture | IA_1_ | 1 | 1 | 1 | 3 | 1 | 1 | 2 | 2 | 3 |
| R20762 | Pus (neck lump) | IA_1_ | 1 | 1 | 1 | 3 | 1 | 1 | 2 | 2 | 3 |
| PRP-002 | Acne | IA_1_ | 1 | 1 | 1 | 3 | 1 | 1 | 2 | 2 | 3 |
| PRP-053 | Acne | IA_1_ | 1 | 1 | 1 | 3 | 1 | 1 | 2 | 2 | 3 |
| PRP-101 | Acne | IA_1_ | 1 | 1 | 1 | 3 | 1 | 1 | 2 | 2 | 3 |
| 157256 | Lagophtalmus | IA_1_ | 1 | 1 | 1 | 3 | 1 | 1 | 2 | 2 | 3 |
| HL099PA1 | Acne | IA_1_ | 1 | 1 | 1 | 3 | 1 | 1 | 2 | 2 | 3 |
| HL096PA2 | Skin | IA_1_ | 1 | 1 | 1 | 3 | 1 | 1 | 2 | 2 | 3 |
| HL043PA1 | Acne | IA_1_ | 1 | 1 | 1 | 3 | 1 | 1 | 2 | 2 | 3 |
| HL007PA1 | Skin | IA_1_ | 1 | 1 | 1 | 3 | 1 | 1 | 2 | 2 | 3 |
| HL056PA1 | Skin | IA_1_ | 1 | 1 | 1 | 3 | 1 | 1 | 2 | 2 | 3 |
| HL074PA1 | Skin | IA_1_ | 1 | 1 | 1 | 3 | 1 | 1 | 2 | 2 | 3 |
| HL043PA2 | Acne | IA_1_ | 1 | 1 | 1 | 3 | 1 | 1 | 2 | 2 | 3 |
| HL053PA1 | Acne | IA_1_ | 1 | 1 | 1 | 3 | 1 | 1 | 2 | 2 | 3 |
| HL083PA1 | Acne | IA_1_ | 1 | 1 | 1 | 3 | 1 | 1 | 2 | 2 | 3 |
| 15614 | Blood | IA_1_ | 1 | 1 | 1 | 3 | 1 | 1 | 2 | 2 | 3 |
| 26048 | Blood | IA_1_ | 1 | 1 | 1 | 3 | 1 | 1 | 2 | 2 | 3 |
| R191618 | Cadaveric skin | IA_1_ | 1 | 1 | 1 | 3 | 1 | 1 | 8 | 6 | 4 |
| P.acn38 | Aqueous humour | IA_1_ | 1 | 1 | 1 | 3 | 1 | 1 | 8 | 6 | 4 |
| P.acn13 | Corneal scrape | IA_1_ | 1 | 1 | 1 | 3 | 1 | 1 | 8 | 6 | 4 |
| P.acn25 | Corneal scrape | IA_1_ | 1 | 1 | 1 | 3 | 1 | 1 | 8 | 6 | 4 |
| 2703 | Cancerous prostate | IA_1_ | 1 | 1 | 1 | 3 | 1 | 1 | 8 | 6 | 4 |
| HL092PA1 | Acne | IA_1_ | 1 | 1 | 1 | 3 | 1 | 1 | 8 | 6 | 4 |
| HL025PA1 | Skin | IA_1_ | 1 | 1 | 1 | 3 | 1 | 1 | 8 | 6 | 4 |
| HL086PA1 | Skin | IA_1_ | 1 | 1 | 1 | 3 | 1 | 1 | 8 | 6 | 4 |
| HL110PA1 | Acne | IA_1_ | 1 | 1 | 1 | 3 | 1 | 1 | 8 | 6 | 4 |
| HL053PA2 | Acne | IA_1_ | 1 | 1 | 1 | 3 | 1 | 1 | 8 | 6 | 4 |
| 3.4.L2 | [16] | IA_1_ | 1 | 1 | 1 | 3 | 1 | 1 | 8 | 6 | 4 |
| 1.5.L1 | [16] | IA_1_ | 1 | 1 | 1 | 3 | 1 | 1 | 8 | 6 | 4 |
| 30.2.L1 | [16] | IA_1_ | 1 | 1 | 1 | 3 | 1 | 1 | 8 | 6 | 4 |
| 24.1.A1 | [16] | IA_1_ | 1 | 1 | 1 | 3 | 1 | 1 | 8 | 6 | 4 |
| 16.2.R1 | [16] | IA_1_ | 1 | 1 | 1 | 3 | 1 | 1 | 8 | 6 | 4 |
| A10 | Acne | IA_1_ | 1 | 1 | 1 | 3 | 1 | 1 | 8 | 6 | 4 |
| 82964 | Wound | IA_1_ | 1 | 1 | 1 | 3 | 1 | 1 | 8 | 6 | 4 |
| 94167 | Abscess | IA_1_ | 1 | 1 | 1 | 3 | 1 | 1 | 8 | 6 | 4 |
| 28585 | Blood | IA_1_ | 1 | 1 | 1 | 3 | 1 | 1 | 8 | 6 | 4 |
| 6-2-4 | Acne | IA_1_ | 1 | 1 | 1 | 3 | 1 | 1 | 8 | 6 | 4 |
| Asn2 | Spinal disc material | IB | 1 | 1 | 1 | 4 | 1 | 4 | 8 | 6 | 5 |
| Asn3 | Spinal disc material | IB | 1 | 1 | 1 | 4 | 1 | 4 | 8 | 6 | 5 |
| R18466 | Neck lymph node | IB | 1 | 1 | 1 | 4 | 1 | 4 | 8 | 6 | 5 |
| KPA171202^d^ | Plate contaminant | IB | 1 | 1 | 1 | 4 | 1 | 4 | 8 | 6 | 5 |
| W1392 | Dental | IB | 1 | 1 | 1 | 4 | 1 | 4 | 8 | 6 | 5 |
| W1998 | Dental | IB | 1 | 1 | 1 | 4 | 1 | 4 | 8 | 6 | 5 |
| P.acn20 | Corneal scrape | IB | 1 | 1 | 1 | 4 | 1 | 4 | 8 | 6 | 5 |
| P.acn32 | Aqueous humour | IB | 1 | 1 | 1 | 4 | 1 | 4 | 8 | 6 | 5 |
| P.acn37 | Eye | IB | 1 | 1 | 1 | 4 | 1 | 4 | 8 | 6 | 5 |
| P.acn40 | Corneal scrape | IB | 1 | 1 | 1 | 4 | 1 | 4 | 8 | 6 | 5 |
| CK17 | Prosthetic hip joint^e^ | IB | 1 | 1 | 1 | 4 | 1 | 4 | 8 | 6 | 5 |
| LED2 | Prosthetic hip joint^e^ | IB | 1 | 1 | 1 | 4 | 1 | 4 | 8 | 6 | 5 |
| RM9 | Surgical skin wound^f^ | IB | 1 | 1 | 1 | 4 | 1 | 4 | 8 | 6 | 5 |
| 6609^b^ | Skin | IB | 1 | 1 | 1 | 4 | 1 | 4 | 8 | 6 | 5 |
| 56 | Cancerous prostate | IB | 1 | 1 | 1 | 4 | 1 | 4 | 8 | 6 | 5 |
| 2937 | Cancerous prostate | IB | 1 | 1 | 1 | 4 | 1 | 4 | 8 | 6 | 5 |
| 3148 | Cancerous prostate | IB | 1 | 1 | 1 | 4 | 1 | 4 | 8 | 6 | 5 |
| HL030PA1 | Skin | IB | 1 | 1 | 1 | 4 | 1 | 4 | 8 | 6 | 5 |
| 19H | Skin | IB | 1 | 1 | 1 | 4 | 1 | 4 | 8 | 6 | 5 |
| GN-3^c^ | Acne | IB | 1 | 1 | 1 | 4 | 1 | 4 | 8 | 6 | 5 |
| PA1^c^ | Acne | IB | 1 | 1 | 1 | 4 | 1 | 4 | 8 | 6 | 5 |
| VA2/8 | Skin | IB | 1 | 1 | 1 | 4 | 1 | 4 | 8 | 6 | 5 |
| 51318 | Skin | IB | 1 | 1 | 1 | 4 | 1 | 4 | 8 | 6 | 5 |
| M27415 | Intravascular catheter | IB | 1 | 1 | 1 | 4 | 1 | 4 | 8 | 6 | 5 |
| 28764 | Intravascular catheter | IB | 1 | 1 | 1 | 4 | 1 | 4 | 8 | 6 | 5 |
| 31412 | Intravascular catheter | IB | 1 | 1 | 1 | 4 | 1 | 4 | 8 | 6 | 5 |
| 59777 | Intravascular catheter | IB | 1 | 1 | 1 | 4 | 1 | 4 | 8 | 6 | 5 |
| 59782 | Intravascular catheter | IB | 1 | 1 | 1 | 4 | 1 | 4 | 8 | 6 | 5 |
| 2673 | Intravascular catheter | IB | 1 | 1 | 1 | 4 | 1 | 4 | 8 | 6 | 5 |
| 887674 | Skin | IB | 1 | 1 | 1 | 4 | 1 | 4 | 8 | 6 | 5 |
| CCUG32901^g^ | Blood | IB | 1 | 1 | 1 | 4 | 1 | 4 | 8 | 6 | 5 |
| 36.1.A1 | [16] | IB | 1 | 1 | 1 | 4 | 1 | 4 | 8 | 6 | 5 |
| 89071 | Wound | IB | 1 | 1 | 1 | 4 | 1 | 4 | 8 | 6 | 5 |
| 96088 | Skin | IB | 1 | 1 | 1 | 4 | 1 | 4 | 8 | 6 | 5 |
| 102778 | Joint aspirate | IB | 1 | 1 | 1 | 4 | 1 | 4 | 8 | 6 | 5 |
| 1945 | Pleural aspirate | IB | 1 | 1 | 1 | 4 | 1 | 4 | 8 | 6 | 5 |
| 4837 | Surgical sample | IB | 1 | 1 | 1 | 4 | 1 | 4 | 8 | 6 | 5 |
| 10738 | Abscess | IB | 1 | 1 | 1 | 4 | 1 | 4 | 8 | 6 | 5 |
| 14179 | Blood | IB | 1 | 1 | 1 | 4 | 1 | 4 | 8 | 6 | 5 |
| 16703 | Otitis media | IB | 1 | 1 | 1 | 4 | 1 | 4 | 8 | 6 | 5 |
| 20527 | Blood | IB | 1 | 1 | 1 | 4 | 1 | 4 | 8 | 6 | 5 |
| 23766 | Wound | IB | 1 | 1 | 1 | 4 | 1 | 4 | 8 | 6 | 5 |
| 24728 | Wound | IB | 1 | 1 | 1 | 4 | 1 | 4 | 8 | 6 | 5 |
| 45808 | Intrauterine device | IB | 1 | 1 | 1 | 4 | 1 | 4 | 8 | 6 | 5 |
| 51056 | Intraoral | IB | 1 | 1 | 1 | 4 | 1 | 4 | 8 | 6 | 5 |
| 58828 | Conjunctiva | IB | 1 | 1 | 1 | 4 | 1 | 4 | 8 | 6 | 5 |
| 57826 | Conjunctiva | IB | 1 | 1 | 1 | 4 | 1 | 4 | 8 | 6 | 5 |
| 61489 | Appendicitis | IB | 1 | 1 | 1 | 4 | 1 | 4 | 8 | 6 | 5 |
| 1-3-1 | Skin | IB | 1 | 1 | 1 | 4 | 1 | 4 | 8 | 6 | 5 |
| 2-4-5 | Skin | IB | 1 | 1 | 1 | 4 | 1 | 4 | 8 | 6 | 5 |
| 4-2-8 | Skin | IB | 1 | 1 | 1 | 4 | 1 | 4 | 8 | 6 | 5 |
| 4-4-1 | Skin | IB | 1 | 1 | 1 | 4 | 1 | 4 | 8 | 6 | 5 |
| 6-3-1 | Skin | IB | 1 | 1 | 1 | 4 | 1 | 4 | 8 | 6 | 5 |
| 7-2-6 | Skin | IB | 1 | 1 | 1 | 4 | 1 | 4 | 8 | 6 | 5 |
| 2986 | Cancerous prostate | II | 17 | 4 | 2 | 4 | 2 | 3 | 10 | 10 | 6 |
| HL060PA1 | Acne | II | 17 | 4 | 2 | 4 | 2 | 3 | 10 | 10 | 6 |
| HL082PA2 | Acne | II | 17 | 4 | 2 | 4 | 2 | 3 | 10 | 10 | 6 |
| Asn5 | Prosthetic hip joint^e^ | II | 17 | 4 | 2 | 4 | 2 | 3 | 10 | 10 | 6 |
| JP1B | Prosthetic hip joint^e^ | II | 15 | 4 | 2 | 4 | 2 | 3 | 10 | 10 | 7 |
| HL110PA3 | Acne | II | 15 | 4 | 2 | 4 | 2 | 3 | 10 | 10 | 7 |
| HL110PA4 | Acne | II | 15 | 4 | 2 | 4 | 2 | 3 | 10 | 10 | 7 |
| Asn8 | Acne | II | 15 | 4 | 2 | 4 | 2 | 3 | 10 | 10 | 7 |
| 72995 | Surgical sample | II | 15 | 4 | 2 | 4 | 2 | 3 | 10 | 10 | 7 |
| 44073 | Blood | II | 15 | 4 | 2 | 4 | 2 | 3 | 10 | 10 | 7 |
| 65763 | Blood | II | 15 | 4 | 2 | 4 | 2 | 3 | 10 | 10 | 7 |
| PV93 | Acne | IA_1_ | 1 | 1 | 1 | 3 | 1 | 1 | 3 | 1 | 8 |
| P135 | Acne | IA_1_ | 1 | 1 | 1 | 3 | 1 | 1 | 3 | 1 | 8 |
| W891 | Dental | IA_1_ | 1 | 1 | 1 | 3 | 1 | 1 | 3 | 1 | 8 |
| HL096PA3 | Skin | IA_1_ | 1 | 1 | 1 | 3 | 1 | 1 | 3 | 1 | 8 |
| HL020PA1 | Acne | IA_1_ | 1 | 1 | 1 | 3 | 1 | 1 | 4 | 1 | 9 |
| HL038PA1 | Acne | IA_1_ | 1 | 1 | 1 | 3 | 1 | 1 | 2 | 4 | 10 |
| HL005PA1 | Skin | IA_1_ | 1 | 1 | 1 | 3 | 1 | 1 | 5 | 2 | 11 |
| M5 | Abscess | IB | 1 | 1 | 1 | 4 | 1 | 1 | 8 | 31 | 12 |
| HL082PA1 | Acne | IA_1_ | 1 | 1 | 1 | 3 | 1 | 1 | 7 | 6 | 13 |
| HL078PA1 | Skin | IA_1_ | 1 | 1 | 1 | 3 | 1 | 14 | 2 | 3 | 14 |
| HL005PA2 | Skin | IA_1_ | 1 | 1 | 10 | 3 | 1 | 1 | 6 | 1 | 15 |
| HL005PA3 | Skin | IA_1_ | 1 | 1 | 10 | 3 | 1 | 1 | 1 | 1 | 16 |
| HL045PA1 | Acne | IA_1_ | 1 | 10 | 1 | 3 | 1 | 1 | 2 | 2 | 17 |
| SK137 | Skin | IA_1_ | 8 | 1 | 1 | 3 | 1 | 1 | 2 | 5 | 18 |
| SK187 | Skin | IA_1_ | 16 | 1 | 1 | 15 | 1 | 4 | 8 | 6 | 19 |
| PRP-60 | Acne | IA_1_ | 5 | 1 | 1 | 3 | 1 | 1 | 1 | 1 | 20 |
| PRP-72 | Acne | IA_1_ | 5 | 1 | 1 | 3 | 1 | 1 | 1 | 1 | 20 |
| P.acn29 | Corneal scrape | IA_1_ | 5 | 1 | 1 | 3 | 1 | 1 | 1 | 1 | 20 |
| VA1/22 | Skin | IA_1_ | 5 | 1 | 1 | 3 | 1 | 1 | 1 | 1 | 20 |
| VA2/9O | Skin | IA_1_ | 5 | 1 | 1 | 3 | 1 | 1 | 1 | 1 | 20 |
| 266 | Pleuropulmonary | IA_1_ | 5 | 1 | 1 | 3 | 1 | 1 | 1 | 1 | 20 |
| 434 | Acne | IA_1_ | 5 | 1 | 1 | 3 | 1 | 1 | 1 | 1 | 20 |
| R19606 | Brain abscess | IA_1_ | 1 | 1 | 1 | 1 | 1 | 1 | 8 | 6 | 21 |
| 2972 | Cancerous prostate | IA_1_ | 1 | 1 | 1 | 1 | 1 | 1 | 8 | 6 | 21 |
| HL110PA2 | Acne | IA_1_ | 1 | 1 | 1 | 1 | 1 | 1 | 8 | 6 | 21 |
| 39.1.A1 | [16] | IA_1_ | 1 | 1 | 1 | 1 | 1 | 1 | 8 | 6 | 21 |
| 6.1.A1 | [16] | IA_1_ | 1 | 1 | 1 | 1 | 1 | 1 | 8 | 6 | 21 |
| P.acn17 | Corneal scrape | IA_2_ | 1 | 1 | 1 | 5 | 3 | 5 | 8 | 7 | 22 |
| P.acn18 | Corneal scrape | IA_2_ | 1 | 1 | 1 | 5 | 3 | 5 | 8 | 7 | 22 |
| M32390 | Intravascular catheter | IA_2_ | 1 | 1 | 1 | 5 | 3 | 5 | 8 | 7 | 22 |
| 10S | Skin | IA_2_ | 1 | 1 | 1 | 5 | 3 | 5 | 8 | 7 | 22 |
| 12C | Skin | IA_2_ | 1 | 1 | 1 | 5 | 3 | 5 | 8 | 7 | 22 |
| HL030PA2 | Skin | IA_2_ | 1 | 1 | 1 | 5 | 3 | 5 | 8 | 7 | 22 |
| 20.2.R1 | [16] | IA_2_ | 1 | 1 | 1 | 5 | 3 | 5 | 8 | 7 | 22 |
| HL063PA2 | Skin | IA_2_ | 1 | 8 | 1 | 5 | 3 | 5 | 8 | 8 | 23 |
| HL067PA1 | Acne | IA_2_ | 1 | 1 | 1 | 5 | 1 | 4 | 8 | 9 | 24 |
| HL103PA1 | Acne | II | 17 | 9 | 2 | 4 | 2 | 3 | 10 | 10 | 25 |
| 65311 | Blood | II | 17 | 9 | 2 | 4 | 2 | 3 | 10 | 10 | 25 |
| HL050PA2 | Skin | II | 17 | 4 | 2 | 17 | 2 | 3 | 11 | 11 | 26 |
| ATCC11828^h^ | Subcutaneous abscess | II | 17 | 4 | 2 | 4 | 9 | 12 | 10 | 13 | 27 |
| J139 | Skin | II | 17 | 4 | 2 | 16 | 2 | 12 | 10 | 12 | 28 |
| VA2/9N | Skin | II | 17 | 4 | 2 | 16 | 2 | 12 | 10 | 12 | 28 |
| VA3/3 | Skin | II | 17 | 4 | 2 | 16 | 2 | 12 | 10 | 12 | 28 |
| VA3/15 | Skin | II | 17 | 4 | 2 | 16 | 2 | 12 | 10 | 12 | 28 |
| VA3/19 | Skin | II | 17 | 4 | 2 | 16 | 2 | 12 | 10 | 12 | 28 |
| VA3/20 | Skin | II | 17 | 4 | 2 | 16 | 2 | 12 | 10 | 12 | 28 |
| SK182 | Skin | IA_1_ | 1 | 13 | 1 | 3 | 1 | 1 | 2 | 5 | 29 |
| HL001PA1 | Skin | II | 17 | 4 | 2 | 4 | 2 | 6 | 10 | 12 | 30 |
| 76618 | Blood | II | 17 | 4 | 2 | 4 | 2 | 6 | 10 | 12 | 30 |
| 6167 | Blood | II | 17 | 4 | 2 | 4 | 2 | 6 | 10 | 12 | 30 |
| 226 | Acne | IA_1_ | 1 | 1 | 1 | 3 | 1 | 1 | 2 | 3 | 31 |
| 12S | Skin | III | 7 | 6 | 3 | 7 | 5 | 9 | 12 | 15 | 32 |
| Asn11 | Spinal disc material | III | 7 | 6 | 3 | 7 | 5 | 9 | 13 | 16 | 33 |
| Asn31 | Spinal disc material | III | 7 | 6 | 3 | 7 | 5 | 9 | 13 | 16 | 33 |
| Asn33 | Spinal disc material | III | 7 | 6 | 3 | 7 | 5 | 9 | 13 | 16 | 33 |
| Asn38 | Spinal disc material | III | 7 | 6 | 3 | 7 | 5 | 9 | 13 | 16 | 33 |
| Asn72 | Spinal disc material | III | 7 | 6 | 3 | 7 | 5 | 9 | 13 | 16 | 33 |
| Asn82 | Spinal disc material | III | 7 | 6 | 3 | 7 | 5 | 9 | 13 | 16 | 33 |
| Asn83 | Spinal disc material | III | 7 | 6 | 3 | 7 | 5 | 9 | 13 | 16 | 33 |
| VA2/4 | Skin | III | 7 | 6 | 3 | 7 | 5 | 9 | 13 | 16 | 33 |
| VA2/5 | Skin | III | 7 | 6 | 3 | 7 | 5 | 9 | 13 | 16 | 33 |
| VA2/14 | Skin | III | 7 | 6 | 3 | 7 | 5 | 9 | 13 | 16 | 33 |
| Asn12 | Spinal disc material | III | 7 | 6 | 3 | 7 | 5 | 9 | 13 | 16 | 33 |
| 15.2.R1 | [16] | IA_1_ | 1 | 1 | 1 | 1 | 1 | 1 | 8 | 22 | 34 |
| R18395 | Kidney | IA_1_ | 1 | 2 | 1 | 2 | 1 | 1 | 2 | 2 | 35 |
| P.acn31 | Aqueous humour | IA_2_ | 1 | 1 | 1 | 13 | 1 | 4 | 8 | 2 | 36 |
| R18544 | Blood culture | IA_1_ | 1 | 1 | 1 | 3 | 1 | 2 | 15 | 1 | 37 |
| PV44 | Acne | IA_1_ | 1 | 1 | 1 | 3 | 1 | 1 | 16 | 1 | 38 |
| W3875 | Dental | IA_1_ | 1 | 1 | 1 | 3 | 1 | 1 | 2 | 20 | 39 |
| P.acn16 | Corneal scrape | IA_1_ | 1 | 5 | 1 | 3 | 1 | 1 | 2 | 2 | 40 |
| P.acn27 | Aqueous humour | IA_1_ | 1 | 5 | 1 | 3 | 1 | 1 | 2 | 2 | 40 |
| P.acn21 | Corneal scrape | IA_1_ | 1 | 1 | 1 | 6 | 1 | 1 | 1 | 1 | 41 |
| PRP-102 | Acne | IB | 1 | 1 | 1 | 4 | 1 | 4 | 8 | 21 | 42 |
| 74873 | Blood | IB | 1 | 1 | 1 | 4 | 1 | 4 | 8 | 21 | 42 |
| 67060 | Wound | IB | 1 | 1 | 1 | 4 | 1 | 4 | 8 | 21 | 42 |
| PRP-003 | Acne | IA_1_ | 8 | 1 | 1 | 3 | 1 | 1 | 2 | 2 | 43 |
| PV77 | Acne | IA_1_ | 11 | 1 | 1 | 3 | 1 | 1 | 1 | 1 | 44 |
| P6 | Acne | IA_1_ | 1 | 1 | 6 | 3 | 1 | 1 | 1 | 1 | 45 |
| P9 | Acne | IA_1_ | 1 | 1 | 6 | 3 | 1 | 1 | 1 | 1 | 45 |
| R17644 | Neck lymph node | IA_1_ | 1 | 1 | 1 | 14 | 1 | 1 | 3 | 24 | 46 |
| P.acn22 | Corneal scrape | IA_1_ | 4 | 1 | 1 | 3 | 1 | 1 | 1 | 1 | 47 |
| R19133 | Bone (tibia) | IA_1_ | 1 | 3 | 1 | 2 | 1 | 1 | 1 | 1 | 48 |
| PRP-62 | Acne | IA_1_ | 1 | 1 | 1 | 9 | 1 | 1 | 1 | 1 | 49 |
| PV139 | Acne | IA_1_ | 12 | 1 | 1 | 3 | 1 | 1 | 1 | 1 | 50 |
| Asn1 | Prosthetic hip joint^e^ | IB | 1 | 1 | 9 | 4 | 1 | 11 | 8 | 25 | 51 |
| Asn7 | Spinal disc material | IA_1_ | 1 | 1 | 1 | 3 | 8 | 1 | 8 | 6 | 52 |
| 3145 | Cancerous prostate | IB | 1 | 1 | 9 | 4 | 1 | 4 | 8 | 6 | 53 |
| 4-2-6 | Skin | IB | 1 | 1 | 9 | 4 | 1 | 4 | 8 | 6 | 53 |
| AT1 | Prosthetic hip joint | IA_1_ | 1 | 1 | 1 | 3 | 1 | 13 | 18 | 1 | 54 |
| GN-1^c^ | Acne | IA_1_ | 1 | 12 | 1 | 3 | 1 | 1 | 8 | 6 | 55 |
| 21.1.L1 | [16] | IB | 1 | 1 | 12 | 4 | 1 | 4 | 8 | 6 | 56 |
| 25.1.R1 | [16] | IA_2_ | 1 | 1 | 1 | 18 | 1 | 4 | 8 | 2 | 57 |
| 37J+ | Skin | IA_2_ | 1 | 1 | 1 | 18 | 1 | 4 | 8 | 2 | 57 |
| R18473 | Blood culture | II | 2 | 4 | 2 | 4 | 2 | 3 | 10 | 26 | 58 |
| RB1B | Prosthetic hip joint^e^ | II | 13 | 4 | 2 | 4 | 2 | 3 | 10 | 10 | 59 |
| P.acn19 | Corneal scrape | II | 3 | 4 | 2 | 4 | 2 | 3 | 10 | 10 | 60 |
| PRP-47 | Acne | II | 1 | 4 | 2 | 4 | 2 | 3 | 10 | 10 | 61 |
| KC1 | Prosthetic hip joint^e^ | II | 3 | 4 | 2 | 10 | 4 | 6 | 10 | 18 | 62 |
| ED1 | Prosthetic hip joint^e^ | II | 14 | 4 | 2 | 4 | 4 | 6 | 10 | 18 | 63 |
| P.acn30 | Vitreous humour | II | 6 | 4 | 2 | 4 | 4 | 6 | 19 | 12 | 64 |
| 2755 | Cancerous prostate | II | 17 | 4 | 2 | 4 | 2 | 3 | 10 | 19 | 65 |
| ATB1 | Prosthetic hip joint^e^ | II | 17 | 4 | 2 | 4 | 2 | 3 | 10 | 19 | 65 |
| RM4 | Prosthetic hip joint^e^ | II | 17 | 4 | 2 | 4 | 2 | 3 | 10 | 19 | 65 |
| M67994 | Intravascular catheter | II | 17 | 4 | 2 | 4 | 2 | 3 | 10 | 11 | 66 |
| 3100 | Cancerous prostate | II | 17 | 4 | 2 | 4 | 4 | 6 | 10 | 17 | 67 |
| P24 | Acne | II | 17 | 4 | 2 | 4 | 4 | 6 | 10 | 17 | 67 |
| TFJ2 | Prosthetic hip joint^e^ | II | 17 | 4 | 2 | 4 | 4 | 6 | 10 | 18 | 68 |
| ET1 | Prosthetic hip joint^e^ | II | 17 | 4 | 2 | 4 | 4 | 6 | 10 | 18 | 68 |
| M45709 | Intravascular catheter | II | 17 | 4 | 2 | 4 | 4 | 6 | 10 | 12 | 69 |
| 26N | Skin | II | 17 | 4 | 2 | 4 | 4 | 6 | 10 | 12 | 69 |
| 29C | Skin | II | 17 | 4 | 2 | 4 | 4 | 6 | 10 | 12 | 69 |
| 28S- | Skin | II | 17 | 4 | 2 | 4 | 4 | 6 | 10 | 12 | 69 |
| PRP-38 | Acne | IC | 9 | 1 | 4 | 8 | 6 | 8 | 14 | 14 | 70 |
| PRP-39 | Acne | IC | 9 | 1 | 4 | 8 | 6 | 8 | 14 | 14 | 70 |
| HL097PA1 | Acne | IC | 9 | 1 | 4 | 8 | 6 | 8 | 14 | 14 | 70 |
| 8F | Skin | II | 18 | 4 | 2 | 4 | 2 | 6 | 20 | 12 | 71 |
| SG2 | Skin | II | 17 | 4 | 2 | 4 | 2 | 12 | 10 | 12 | 72 |
| 5-3-4 | Acne | II | 17 | 4 | 2 | 4 | 2 | 12 | 10 | 12 | 72 |
| Asn13 | Spinal disc material | III | 7 | 6 | 3 | 11 | 5 | 9 | 13 | 16 | 73 |
| Asn85 | Spinal disc material | III | 7 | 6 | 3 | 7 | 5 | 9 | 13 | 27 | 74 |
| 7B | Skin | III | 7 | 6 | 3 | 7 | 5 | 9 | 12 | 23 | 75 |
| 17S | Skin | III | 7 | 6 | 3 | 7 | 5 | 9 | 12 | 23 | 75 |
| 21S | Skin | III | 7 | 6 | 3 | 7 | 5 | 9 | 12 | 23 | 75 |
| 23C | Skin | III | 7 | 6 | 3 | 7 | 5 | 9 | 12 | 23 | 75 |
| 24A | Skin | III | 7 | 6 | 3 | 7 | 5 | 9 | 12 | 23 | 75 |
| 41F | Skin | III | 7 | 6 | 3 | 7 | 5 | 9 | 12 | 23 | 75 |
| 17A | Skin | III | 7 | 6 | 3 | 7 | 5 | 9 | 12 | 23 | 75 |
| 32P | Skin | III | 7 | 6 | 3 | 7 | 5 | 9 | 12 | 23 | 75 |
| 18B | Skin | III | 7 | 6 | 3 | 7 | 10 | 9 | 12 | 16 | 76 |
| 25A | Skin | III | 7 | 6 | 3 | 7 | 10 | 9 | 12 | 16 | 76 |
| 18S | Skin | III | 7 | 6 | 3 | 7 | 10 | 9 | 12 | 16 | 76 |
| 20C | Skin | III | 7 | 6 | 3 | 7 | 5 | 9 | 12 | 16 | 77 |
| 32C | Skin | III | 7 | 6 | 3 | 7 | 5 | 9 | 12 | 16 | 77 |
| 30F | Skin | III | 7 | 6 | 3 | 7 | 5 | 9 | 12 | 16 | 77 |
| VA3/4 | Skin | IB | 1 | 1 | 1 | 4 | 1 | 4 | 8 | 29 | 78 |
| A3 | Skin | II | 17 | 4 | 2 | 4 | 2 | 12 | 10 | 28 | 79 |
| PF4 | Skin | IA_1_ | 1 | 11 | 1 | 3 | 1 | 1 | 8 | 6 | 80 |
| Asn10 | Prosthetic hip joint^e^ | III | 7 | 6 | 7 | 7 | 5 | 9 | 12 | 16 | 81 |
| XnL1 | Fatal head granuloma | IA_1_ | 1 | 1 | 1 | 3 | 1 | 1 | 21 | 2 | 82 |
| Z3^c^ | Acne | IA_1_ | 1 | 1 | 1 | 3 | 1 | 1 | 8 | 30 | 83 |
| 5051 | Intravascular catheter | IB | 19 | 1 | 1 | 4 | 1 | 4 | 8 | 21 | 84 |
| PV66 | Acne | IC | 9 | 1 | 5 | 8 | 6 | 8 | 14 | 6 | 85 |
| PRP-78 | Acne | IA_1_ | 1 | 1 | 1 | 3 | 7 | 1 | 22 | 2 | 86 |
| GP1^c^ | Acne | IA_1_ | 1 | 1 | 11 | 3 | 1 | 1 | 1 | 1 | 87 |
| 413 | Acne | IA_1_ | 1 | 1 | 1 | 3 | 1 | 1 | 17 | 33 | 88 |
| PRP-81 | Acne | IB | 1 | 1 | 1 | 4 | 1 | 4 | 8 | 1 | 89 |
| P.acn36 | Corneal scrape | III | 7 | 6 | 3 | 7 | 5 | 7 | 23 | 32 | 90 |
| HL050PA1 | Skin | IA_2_ | 1 | 1 | 1 | 5 | 1 | 4 | 9 | 2 | 91 |
| A9 | Acne | IA_1_ | 1 | 1 | 1 | 15 | 9 | 13 | 2 | 2 | 92 |
| PF3 | Acne | IA_1_ | 1 | 1 | 1 | 3 | 8 | 1 | 1 | 1 | 93 |
| PF9 | Acne | IA_1_ | 1 | 1 | 1 | 10 | 1 | 1 | 8 | 6 | 94 |
| PF12 | Acne | IA_1_ | 1 | 1 | 1 | 9 | 9 | 1 | 8 | 6 | 95 |
| 12553 | Blood | II | 15 | 4 | 2 | 4 | 2 | 3 | 24 | 10 | 96 |
| M34 | Wound | IA_1_ | 1 | 1 | 1 | 4 | 1 | 1 | 25 | 1 | 97 |
| 101320 | Surgical sample | IB | 1 | 1 | 1 | 4 | 1 | 4 | 8 | 34 | 98 |
| 24763 | Intraabdominal | IB | 1 | 1 | 1 | 4 | 1 | 4 | 26 | 6 | 99 |
| 63848 | Conjunctiva | II | 20 | 4 | 2 | 4 | 2 | 6 | 10 | 12 | 100 |
| 76793 | Oral | IA_1_ | 1 | 1 | 1 | 3 | 1 | 14 | 1 | 1 | 101 |
| M8 | Acne | IA_1_ | 1 | 1 | 1 | 3 | 1 | 14 | 8 | 6 | 102 |
| 19695 | Wound | IA_1_ | 1 | 15 | 1 | 3 | 1 | 1 | 1 | 1 | 103 |
| 44261 | Blood | IA_1_ | 21 | 1 | 1 | 3 | 1 | 1 | 1 | 6 | 104 |
| 56853 | Heart implant | IA_1_ | 1 | 1 | 1 | 3 | 1 | 1 | 1 | 6 | 105 |
| 46361 | Acne | II | 15 | 4 | 2 | 19 | 2 | 3 | 10 | 10 | 106 |
| 5-1-3 | Acne | IC | 9 | 1 | 4 | 8 | 6 | 8 | 14 | 6 | 107 |
| 2-1-7 | Acne | II | 17 | 4 | 2 | 20 | 2 | 12 | 10 | 12 | 108 |
| 6-2-3 | Acne | II | 17 | 4 | 2 | 4 | 2 | 15 | 10 | 12 | 109 |
| 8-4-1 | Acne | IA_2_ | 1 | 1 | 1 | 3 | 1 | 4 | 8 | 2 | 111 |
| IT41EA | Acne | IA_1_ | 1 | 1 | 1 | 3 | 1 | 1 | 27 | 1 | 112 |
| 16J | Skin | II | 18 | 4 | 2 | 4 | 2 | 6 | 20 | 35 | 113 |
| 11G | Skin | III | 7 | 6 | 3 | 7 | 5 | 16 | 12 | 16 | 114 |

^a^Type strain purchased from the National Collection of Type Cultures (Colindale, UK);

^b^Isolates from the study of Nagy et al. [14];

^c^Isolates from the study of Lodes et al. [13];

^d^Strain purchased from the German Collection of Microorganisms and Cell Cultures (DSMZ; Braunschweig, Germany);

^e^Associated with sonicate from failed prosthetic hip implant;

^f^Relates to spinal surgery skin wound;

^g^Strain from Culture Collection, University of Göteborg;

^h^Strain purchased from American Type Culture Collection.
